# Vascular access options for complex high-risk indicated percutaneous coronary interventions: contemporary strategies, trade-offs, and practical algorithms

**DOI:** 10.3389/fcvm.2026.1883827

**Published:** 2026-09-28

**Authors:** Giuseppe Colletti, Chadi Ghafari, Zoltan Ruzsa, Olivier Gach, Pieter-Jan Palmers, Lucien Finianos, Tim Noterdaeme, Augustin Tchassem Dimdie, Quentin Trefois, Adrien Jossart, Laura Peter, Agostino Spanò, Nino Cocco, Marouane Boukhris, Claudiu Ungureanu

**Affiliations:** 1 Department of Cardiology, Clinique St. Joseph—Groupe Vivalia, Arlon, Belgium; 2 Department of Internal Medicine, Hôpital Vivalia De Libramont, Arlon, Belgium; 3 Internal Medicine Department, Cardiology Center, University of Szeged, Szeged, Hungary; 4 Cardiovascular Center, Semmelweis University, Budapest, Hungary; 5 Department of Cardiology, CHC MontLégia, Liège, Belgium; 6 Department of Cardiology, Jolimont Hospital—CHU Helora, La Louvière, Belgium; 7 Department of Cardiology, Centre Hospitalier de Wallonie Picarde, Tournai, Belgium; 8 Department of Cardiovascular Sciences, Campus Bio-medico University Hospital, Rome, Italy; 9 Department of Cardiology, CHU Saint Etienne, Saint-Étienne, France

**Keywords:** calcified PCI, chronic total occlusion, complex high-risk indicated PCI, femoral access, mechanical circulatory support, radial access, vascular access

## Abstract

**Background:**

Vascular access is an important component of CHIP planning that influences procedural feasibility, safety, and completeness. It affects bleeding and vascular complications, guide-catheter support, compatibility with intravascular imaging and calcium-modification devices, chronic total occlusion techniques, closure and bailout options, and mechanical circulatory support (MCS) planning. Contemporary CHIP populations are increasingly older or frail and more often have chronic kidney disease, peripheral artery disease, reduced left ventricular ejection fraction, prior bypass surgery, calcification, or CTO; access choice should extend beyond radial vs. femoral.

**Summary:**

This review appraises proximal and distal radial, femoral, ulnar, and brachial access; sheathless, slender, and RailTracking strategies; large-bore femoral workflows; and access planning when MCS is considered. We discuss preprocedural arterial assessment, ultrasound and micropuncture, prior access footprint, closure feasibility, hostile femoral anatomy, alternative access, and complication prevention and rescue. The available literature supports a non-dogmatic approach in which access architecture is matched to patient vulnerability, arterial anatomy, lesion complexity, guide and device requirements, support needs, haemostasis feasibility, and bailout planning. Recent randomised evidence further reinforces selective rather than routine prophylactic MCS in nonemergent high-risk PCI.

**Conclusions:**

Forearm access should remain central whenever it permits procedural success without compromising device delivery, imaging, guide support, or bailout flexibility. Femoral access remains indispensable when large-bore support, maximal guide stability, complex device delivery, or rapid escalation are anticipated. Within a comprehensive CHIP plan, the preferred access architecture is the smallest one that enables the intended revascularisation, safe haemostasis, and timely rescue.

## Introduction

Contemporary CHIP includes patients with advanced comorbidity, impaired ventricular function, left main or multivessel disease, chronic total occlusions, severe calcification, and limited tolerance of procedural ischaemia (1–4).

Although no uniform international definition or global denominator exists, analyses of the UK BCIS national registry document an expanding high-risk PCI case mix: the proportion of non-emergency procedures with at least one CHIP factor rose from 58.8% in 2006 to 68.6% in 2016, and the proportion with a CHIP score ≥5 more than doubled from 2.5% to 5.3% (5). A complementary England/Wales analysis found that the share of CHIP performed in nonsurgical centres increased from 12.5% in 2006 to 42.0% in 2017 (6).

In this setting, vascular access is not a preliminary technical detail but an enabling component of the broader procedural plan. It can facilitate or constrain guide support, device delivery, intravascular imaging, calcium modification, and MCS, while overall success also depends on patient selection, lesion preparation, haemodynamic assessment, support strategy, and operator and institutional capability (1–4, 7).

This review is organised around two practical pathways: CHIP without planned MCS, in which forearm-first or standard femoral/hybrid access is usually sufficient, and CHIP with planned MCS, in which coronary access, support access, closure, and rescue must be designed together.

Evidence is interpreted hierarchically throughout this review. Randomised comparisons anchor conclusions about efficacy and safety; registries and comparative cohorts inform associations and generalisability; uncontrolled series establish feasibility only; and expert consensus informs workflow where comparative evidence is absent. Evidence derived from ACS, TAVR, peripheral, or structural interventions is treated as indirect when applied to elective CHIP. Formal guideline classes and levels are reported only when explicitly assigned.

## Access planning before puncture

European guidance is clearest at the level of broad access route and clinical setting. The 2023 ESC ACS guideline recommends radial access as the standard approach unless overriding procedural considerations exist (Class I, Level A) (8). The 2024 ESC CCS guideline recommends radial access as the preferred site when invasive coronary angiography is indicated (Class I, Level A) and states that a microaxial flow pump may be considered in selected patients with heart failure with reduced ejection fraction undergoing complex high-risk PCI in experienced centres (Class IIb, Level C) (9). Neither statement mandates radial access for every elective CHIP procedure or large-bore support configuration (8, 9). The 2023 ACS guideline also permits short-term MCS in severe or refractory cardiogenic shock (Class IIb, Level C) but advises against routine IABP in ACS-related shock without mechanical complications (Class III, Level B) (8). The 2025 EAPCI/ESC Working Group and 2021 EAPCI/ACVC documents are ungraded consensus statements that emphasise Heart Team selection, the least invasive haemodynamically adequate support strategy, and institutional competence (1, 10). Where graded guidance is absent, anatomy, procedural requirements, and operator and centre capability remain decisive (1, 10).

Large-bore support magnifies these considerations. Randomised closure evidence and percutaneous ventricular assist device guidance link vascular outcomes to arterial anatomy, sheath profile, insertion and removal requirements, and closure strategy (10–12). Calcified closure zones, prior access injury, peripheral artery disease, and closure-device failure further influence bleeding, limb ischaemia, and bailout (13–15). When iliofemoral anatomy is prohibitive, transaxillary or transcaval routes may be considered only in experienced centres with device-specific imaging, closure, and endovascular or surgical rescue capability; neither is a routine substitute for femoral access (16).

The access plan should integrate patient vulnerability, coronary and procedural complexity, anticipated equipment, arterial anatomy, and rescue options (Figure 1). CTO, complex bifurcation disease, and severe calcium may increase guide, lumen, imaging, and lesion-preparation requirements (17, 18). MCS commonly adds an independent large-bore route (2, 10, 12). Selected single-access strategies are possible (19). The access-planning goal is the smallest architecture that safely enables the broader procedural plan and timely escalation.

**Figure 1:**
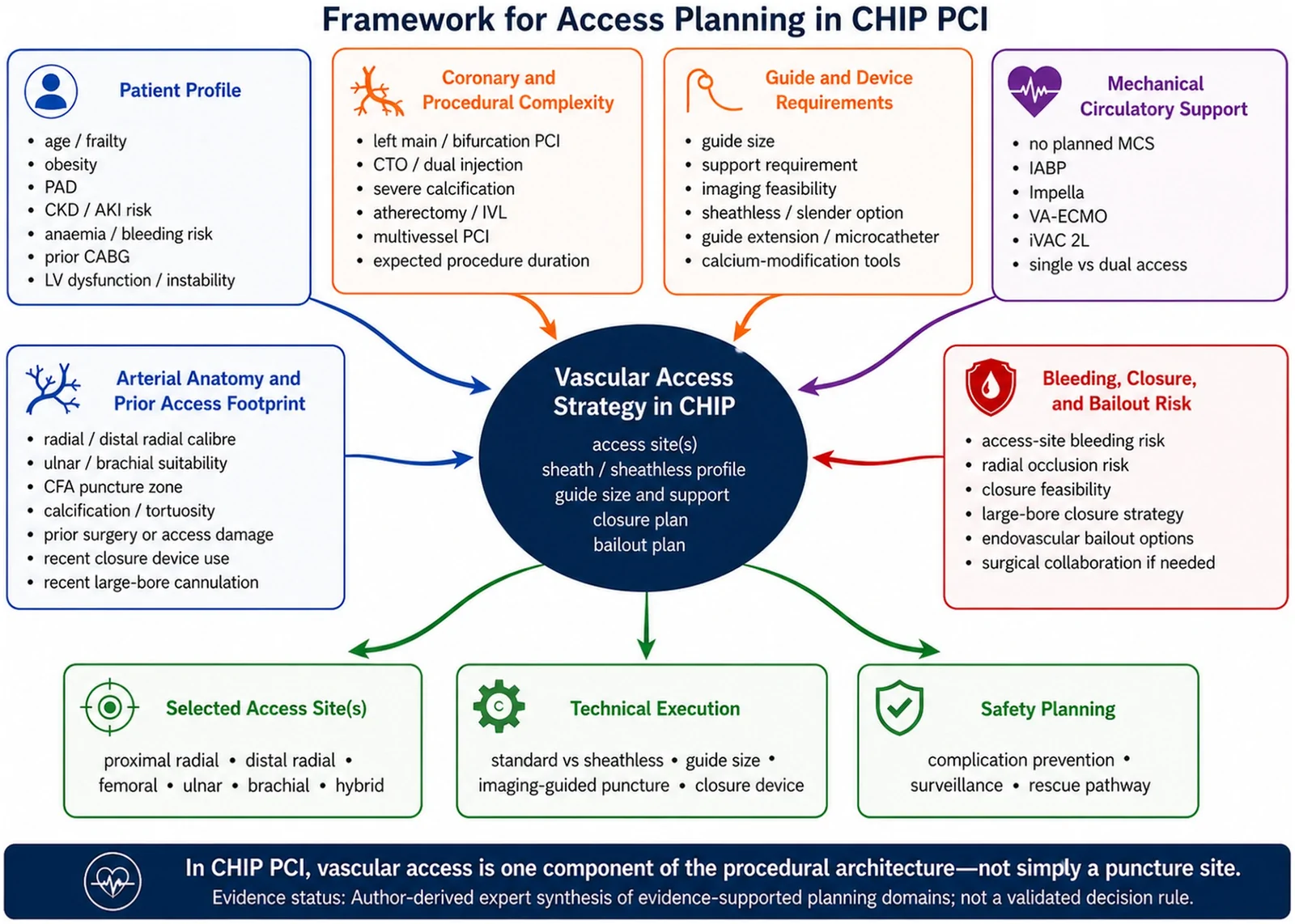
Framework for access planning in CHIP PCI. Conceptual framework for vascular-access planning in complex high-risk indicated percutaneous coronary intervention (CHIP). Access strategy should integrate patient profile, coronary and procedural complexity, guide and device requirements, anticipated need for mechanical circulatory support, arterial anatomy and prior access footprint, and access-site bleeding, closure, and bailout risk. The final strategy should define not only the puncture site, but also sheath or sheathless profile, guide-catheter plan, haemostasis strategy, and rescue pathway. This access-focused framework complements, rather than replaces, planning for lesion preparation, intravascular imaging, calcium modification, haemodynamic assessment, MCS selection, and operator/team capability. Evidence status: This conceptual framework is an author-derived expert synthesis informed by the cited literature. Although the component planning domains are evidence-supported to varying degrees, their integration, arrangement, and relative weighting have not been prospectively validated and do not represent a formal guideline recommendation. AKI, acute kidney injury; CABG, coronary artery bypass grafting; CFA, common femoral artery; CKD, chronic kidney disease; CTO, chronic total occlusion; IABP, intra-aortic balloon pump; IVL, intravascular lithotripsy; LV, left ventricular; MCS, mechanical circulatory support; PAD, peripheral artery disease; PCI, percutaneous coronary intervention; VA-ECMO, veno-arterial extracorporeal membrane oxygenation.

The clinical profile defines tolerance for access-related harm. Advanced age, frailty, anaemia, chronic kidney disease, peripheral artery disease, ventricular dysfunction, prior coronary bypass surgery, and previous access injury or large-bore cannulation all modify bleeding, transfusion, limb-ischaemia, and recovery risk (1, 7, 12, 14, 15). Contemporary Veterans Affairs data illustrate this interaction: surgically ineligible patients undergoing left-main or multivessel PCI had more comorbidity and lesion complexity and more frequent use of multiple arterial access sites, intravascular imaging, atherectomy, and MCS (20).

The procedural profile should be forecast before puncture. Operators should anticipate guide size, intravascular imaging, atherectomy or other calcium-modification tools, dual injection, retrograde CTO work, possible support, and the likelihood of crossover (1, 2, 17, 18). This forecast should be translated into a primary route, a conversion threshold, and a closure and rescue plan (7, 12, 15).

Arterial imaging is central. For femoral access, ultrasound and, when relevant, computed tomography or angiography should define vessel diameter, bifurcation height, calcification, tortuosity, prior repair, and closure-zone suitability (7, 12, 15). FAUST and subsequent individual-participant-data and learning-curve analyses provide randomised and pooled support for routine ultrasound integration; detailed trial outcomes are discussed below (21–23).

Upper-extremity access deserves the same discipline. In RAUST, real-time ultrasound improved first-pass radial cannulation and shortened access time (24). A combined ultrasound-angiographic workflow may identify radial occlusion despite a distal pulse, loops, stenoses, spasm, and other unfavourable anatomy before premature crossover (25).

Closure planning begins before puncture. Micropuncture, common femoral targeting, and preservation of guidewire control until haemostasis is secure are particularly important when large-bore access is possible (7, 12, 15). A secondary route for crossover angiography, balloon tamponade, or covered-stent rescue should be considered before upsizing rather than after closure has failed (15, 26).

## Part I. Chip without planned MCS: forearm-first and standard-access pathways

### Forearm-first access when procedural demands can be met

Radial access remains the default starting point for many CHIP procedures because it reduces access-site bleeding and vascular complications and favours mobilisation and comfort (27–30). The ESC Class I, Level A recommendations support the radial route in ACS and CCS but do not mandate it for every elective CHIP procedure (8, 9). Route selection must still account for artery calibre and patency, guide and device requirements, possible dual access or MCS, and crossover and rescue feasibility (28–30).

The general safety rationale for a forearm-first mindset is reinforced by an individual-patient-data meta-analysis of 21,600 participants from seven randomised trials, in which radial access reduced 30-day all-cause mortality (1.6% vs. 2.1%) and major bleeding (1.5% vs. 2.7%) compared with femoral access (31). Because 95% of participants had ACS, the mortality finding cannot be assumed to apply to elective CHIP, for which direct randomised prognostic evidence is lacking. Direct randomised evidence in complex elective PCI supports fewer access-site complications without impaired procedural success, but has not established a mortality benefit (32).

The main upper-extremity options are compared in Supplementary Figure S1, and the broader access trade-offs are summarised in Table 1.

**Table 1:** Comparative features of the major vascular access strategies used in CHIP.

Access strategy	Main strengths	Main limitations/risks	Most suitable CHIP contexts	Evidence/guidance
Conventional proximal radial	Lower bleeding, comfort/mobility, familiar workflow; preserves femoral bailout/MCS. (27–31)	Limited guide lumen/support unless slender/sheathless; RAO/spasm remain possible. (34–38)	Default when forearm access supports planned equipment. (7, 25, 28)	**Evidence:** RCT/meta-analytic evidence, mainly ACS; elective-CHIP mortality evidence lacking. (27, 31, 32) **Guidance:** ESC radial route: Class I, Level A (ACS/CCS). (8, 9)
Distal radial	Forearm-radial preservation; potential for shorter haemostasis; repeat access. (42–47)	Smaller calibre/more difficult puncture; higher crossover than proximal radial. (43–47)	Radial preservation/repeat access; selected CTO/dual-forearm workflows in experienced centres. (48–51, 53)	**Evidence:** RCT/meta-analytic data show lower RAO but higher crossover; CHIP evidence remains observational/operator-dependent. (43–53, 71) **Guidance:** No formal ESC class; selective expert use. (8, 9)
Ulnar	Alternative when radial access is unsuitable or intentionally preserved. (56–58)	Deeper/harder to compress; haematoma risk and proximity to the ulnar nerve; occlusion may threaten perfusion with ipsilateral RAO. (27, 28, 57–59)	Selective non-femoral alternative. (56–59)	**Evidence:** Limited randomised/observational evidence; anatomy- and operator-dependent. (56–59) **Guidance:** No formal ESC class; selective alternative. (8, 9)
Brachial	Upper-limb rescue when radial/ulnar are unsuitable and femoral undesirable. (60–62)	More bleeding, thrombosis and nerve injury than radial; repair may be needed. (60–62)	Superselected hostile-peripheral/femoral anatomy. (16, 60–62)	**Evidence:** Contemporary evidence is observational (60–62); the only coronary RCT is PTCA-era (120). **Guidance:** No formal ESC class; expert-centre bailout. (8, 9)
Standard femoral	Strong support, easy upsizing, broad compatibility for complex/prolonged PCI. (7, 33, 63, 67)	Higher bleeding/vascular risk than forearm if puncture/closure is suboptimal. (21–23, 27, 31)	Left main, complex bifurcation, selected CTO/support-intensive PCI. (9, 18, 63, 67)	**Evidence:** Randomised data show better puncture metrics; pooled evidence suggests vascular benefit; ULTRACOLOR was neutral. (21–23, 73) **Guidance:** No ESC ultrasound class. (8, 9)
Large-bore femoral	Enables planned MCS/large-device workflows; maximal versatility. (2, 10, 12, 15)	Bleeding, closure failure and endovascular/surgical bailout risk. (11, 12, 14, 15)	Protected PCI, Impella, VA-ECMO, iVAC 2L and other large-bore workflows. (2, 10, 12, 14, 15)	**Evidence:** Observational/consensus; closure RCTs TAVR-derived. (1, 2, 10–15) **Guidance:** Experienced centres; no access-site class. (1, 8–10)
Sheathless/slender forearm	Less sheath–artery mismatch while preserving guide lumen/support. (34–38, 40, 52, 53, 71, 119)	Technically demanding; cannot replace femoral when maximal support is required. (34–38, 40, 52, 71, 119)	Selected CTO, calcium-modification or bifurcation PCI in small radial arteries. (34, 38, 52, 53, 71, 72)	**Evidence:** Limited randomised access and observational complex-PCI data. (34–38, 40, 52, 53, 71, 119) **Guidance:** No ESC class; selective expert use. (8, 9)

CHIP, complex high-risk indicated PCI; MCS, mechanical circulatory support; RAO, radial artery occlusion. Evidence labels are descriptive; only guideline-assigned classes are shown. Radial Class I, Level A applies to the route, not a puncture site; “experienced” denotes feasibility, not superiority.

#### Conventional proximal radial access

Conventional proximal radial access remains the reference upper-extremity strategy in contemporary coronary intervention because its safety and efficacy are supported by the largest body of evidence and the most mature best-practice framework (28–30). For CHIP, its main strengths are its lower bleeding liability compared with femoral access, reproducible haemostasis, operator familiarity, and the possibility of preserving a femoral route for bailout or support-device implantation (7, 28–30).

At the same time, proximal radial access has clear limits in CHIP. Guide support may become suboptimal in heavily calcified lesions, left main or complex bifurcation interventions, and dual-catheter CTO procedures (17, 18, 33). Outer-profile constraints may limit guide size unless slender or sheathless systems are used (34–38). Tracking techniques can facilitate delivery through difficult upper-extremity anatomy (39). These limitations do not invalidate a radial-first philosophy, but they do make it necessary to move from access-site preference to procedural matching.

The evidence base for profile reduction is expanding beyond small series. In a Japanese registry of 259,401 stable elective PCI cases, PCI with a 5 Fr or smaller sheath was associated with shorter procedure time, lower contrast volume, fewer PCI failures, and fewer access-site bleeding complications requiring transfusion than PCI with 6 Fr or larger systems, although selection bias and the non-CHIP population limit direct extrapolation (40). A 2025 meta-analysis of dedicated sheathless vs. sheath-based transradial PCI similarly found less radial spasm and less access-site crossover, with no statistically significant differences in radial artery occlusion, access-related bleeding, or procedural failure (38).

This broader balance was illustrated in a 2024 network meta-analysis comparing femoral, radial, distal radial, and ulnar access for coronary angiography or PCI. Radial access reduced major bleeding compared with femoral access, whereas distal radial and ulnar access had point estimates favouring lower major bleeding with less certain evidence. For access-site haematoma, radial, distal radial, and ulnar access were each associated with lower risk than femoral access; distal radial ranked first for haematoma, while ulnar access was associated with more haematoma than radial access (OR: 1.48, 95% CI: 1.03–2.14) (27).

#### Distal radial access

Distal radial access (dTRA) was developed as a strategy to preserve the forearm radial artery: because the puncture lies beyond important palmar branches, compression or local thrombosis may preserve forearm radial flow, although this remains a physiological rationale rather than evidence of superior clinical outcomes (41, 42).

Randomised evidence demonstrates a trade-off rather than uniform superiority (43, 44). Across 14 trials including 6,208 patients, dTRA reduced radial artery occlusion (RAO) at longest follow-up [1.6% vs. 5.2%; risk ratio (RR) 0.36] and EASY grade II or higher haematoma (RR 0.51), but increased crossover (12.5% vs. 3.8%; RR 3.08), puncture attempts, and access time (45). The absolute RAO reduction may, however, be modest when contemporary proximal-radial best practices keep RAO very low. In DISCO RADIAL, RAO was 0.31% with dTRA and 0.91% with conventional access (*p* = 0.29), whereas crossover and spasm remained more frequent with dTRA despite shorter haemostasis (46). CORRECT RADIAL produced a similar non-significant RAO difference but was stopped after approximately half the planned recruitment and was therefore underpowered (47).

Thus, the practice-changing conclusion is not that dTRA should replace proximal radial access, but that it is a selective preservation strategy when maintaining the forearm radial segment has concrete future value and the operator has sufficient expertise to manage higher puncture and crossover risk. Randomised evidence has not established benefit in major clinical outcomes (43–47). Dedicated complex or large-bore CHIP applications are supported predominantly by observational feasibility studies (48–53). Their clinical use therefore remains hypothesis-generating and centre-dependent (54). Dedicated randomised evidence on forearm RAO after complex large-bore PCI is pending in DISCO COMPLEX (55).

#### Ulnar access

Key evidence includes a large observational ulnar-first series and two large randomised comparisons with radial access (56–58).

Randomised data explain why this role should remain selective. AURA of ARTEMIS, which tested a default transulnar strategy in 902 patients, was stopped after interim analysis; crossover was substantially more frequent with transulnar access, although the cluster-adjusted composite endpoint was inconclusive (57). Conversely, the single-centre AJULAR study, performed by operators with prior ulnar experience, found transulnar access non-inferior to radial access for its composite endpoint; crossover did not differ statistically, whereas non-inferiority for large haematoma was inconclusive (58). The practical message is that ulnar access is a useful reserve strategy only when operator experience and patient anatomy support it.

The largest modern single-centre ulnar-first series reported successful cannulation in 2,495 of 2,525 eligible patients (98.8%), ulnar-route procedure completion in 2,414, local bleeding in 1.6%, acute loss of ulnar pulse in 1.3%, and no access-site gangrene, pseudoaneurysm, arteriovenous fistula, or neurological deficit (56). These results support feasibility in experienced hands but do not make ulnar access equivalent to radial access in evidence maturity or haemostasis simplicity.

Thus, ulnar access should be considered a selective forearm alternative rather than a routine default for CHIP. Its value is greatest when preservation of an upper-extremity strategy matters and the operator is familiar with its deeper course, more difficult compression, and closer relationship to the ulnar nerve (30). When ipsilateral radial artery occlusion is present, contemporary evidence on the vascular safety and hand-function consequences of transulnar access remains limited; the prospective ULNART cohort is designed to evaluate these outcomes with standardised ultrasound, neurological testing, and patient-reported measures (59). Until outcome data are available, ulnar patency and palmar collateral supply warrant careful assessment.

#### Brachial access

Most contemporary brachial-access evidence is observational and derives from peripheral and aortic endovascular practice rather than coronary PCI. In the Ozcinar analysis, overall access complications and haematoma were significantly more frequent after ultrasound-guided percutaneous than open surgical brachial access, while thromboembolic events and reoperations were numerically but not statistically more frequent (60). In ARCHIBAL, technical success was high, but access-site complications were significantly more frequent with percutaneous than open brachial access; complications were also more frequent when vascular closure devices were used and tended to increase with larger sheath size (61). These data support brachial access only as a superselected strategy, with early surgical involvement when sheath size or artery quality raises concern.

These results should not be extrapolated uncritically to coronary PCI, but they are informative for CHIP. They suggest that brachial access may still have a role in carefully selected anatomies, especially when a more proximal upper-extremity route is required, but that open exposure may be safer than a purely percutaneous strategy when sheath size increases or when the artery is small or diseased (60, 61). For this reason, brachial access should be treated as a superselected strategy rather than a routine third forearm option.

Coronary-specific contemporary data support this cautious positioning. In the BCIS National PCI Audit, brachial access accounted for only 1,133 of 861,773 procedures (0.13%), and its use was associated with peripheral vascular disease, rotational atherectomy, chronic renal disease, left main PCI, prior CABG, and older age (62). Adjusted outcomes were worse than with radial or femoral access overall, whereas among default radial operators brachial and femoral outcomes were broadly similar except for more arterial complications with brachial access (62). These observational data support brachial access only as an experienced-centre bailout route for selected patients without usable radial or femoral options. Older coronary comparisons were conducted in the PTCA era, and no contemporary randomised CHIP evidence establishes comparative safety or efficacy (30, 62).

#### Dual forearm and hybrid upper-extremity strategies

Upper-extremity access can be expanded into an access array rather than abandoned when the first puncture site is suboptimal. CTO registries and randomised studies show why dual-catheter procedures may require bilateral forearm or radial-femoral planning (33, 63–67), while distal radial, ulnar, and other alternative forearm routes can support selected CTO cases (48–51). Most evidence for these alternative configurations is observational, and guide requirements, operator experience, and a predefined conversion threshold remain decisive.

## Sheathless and slender strategies

Sheathless and slender systems help separate the inner lumen required for complex PCI from the outer profile that must be accommodated by the artery (34–38, 68). This consideration is particularly relevant to the radial and distal radial routes, where calibre is often smaller (69, 70). These systems should be regarded as equipment-enabling strategies rather than a reason to force forearm access. In a randomised comparison of a 6.5 Fr sheathless Eaucath with a 6.0 Fr Glidesheath Slender, the sheath-to-artery ratio and the composite of radial artery occlusion plus symptomatic spasm were lower with the sheathless system, although neither component was individually significant (36). Comparative evidence therefore supports improved vascular compatibility, not superior clinical outcomes.

Evidence in complex PCI is otherwise predominantly observational. Multicentre registries and small series indicate that 6.5–8 Fr sheathless or slender forearm platforms can support bifurcation, CTO, rotational atherectomy, and other complex PCI, with high procedural success and low reported access-complication rates in selected patients (34, 35, 37, 52, 53). These studies establish feasibility rather than superiority: patient and operator selection, small samples, non-randomised comparisons or absent concurrent controls, and incomplete or non-systematic radial-occlusion surveillance limit generalisability.

RailTracking and Distal RailTracking (DRT) extend the same principle by facilitating advancement of larger guiding catheters without the external profile of a conventional introducer sheath (39, 71). In the observational SWITCH D-RIL registry, adoption of DRT was associated with greater use of large-bore guides, larger burrs, and adjunctive calcium-modification devices, with similar short-term procedural and clinical outcomes (71). This suggests that DRT may expand the lesion-preparation options available from the forearm, but it does not establish comparative safety or clinical benefit; the evidence remains hypothesis-generating, operator-dependent, and derived from experienced centres.

The practical implication is that access should be chosen for the entire lesion-preparation plan. A sheathless or slender strategy is reasonable when the required guide support, intravascular imaging, calcium modification, and device compatibility can be delivered without repeated compromise or avoidable crossover. When those requirements exceed a safe forearm platform, planned femoral or hybrid access is preferable (17, 34–40, 71, 72).

Detailed scenario-specific recommendations are provided in Supplementary Appendix 1 and Table S1.

## Standard femoral or hybrid access when forearm access is insufficient

Femoral access remains indispensable when maximal guide support, larger inner-lumen guiding catheters, complex device delivery, prolonged instrumentation, or dual-catheter work are anticipated (2, 7, 33). It should be selected deliberately rather than only after forearm failure, with puncture, closure, and rescue planned together (7, 12, 15).

A structured workflow for standard and large-bore femoral access planning, puncture, closure, and rescue is proposed in Figure 2.

**Figure 2:**
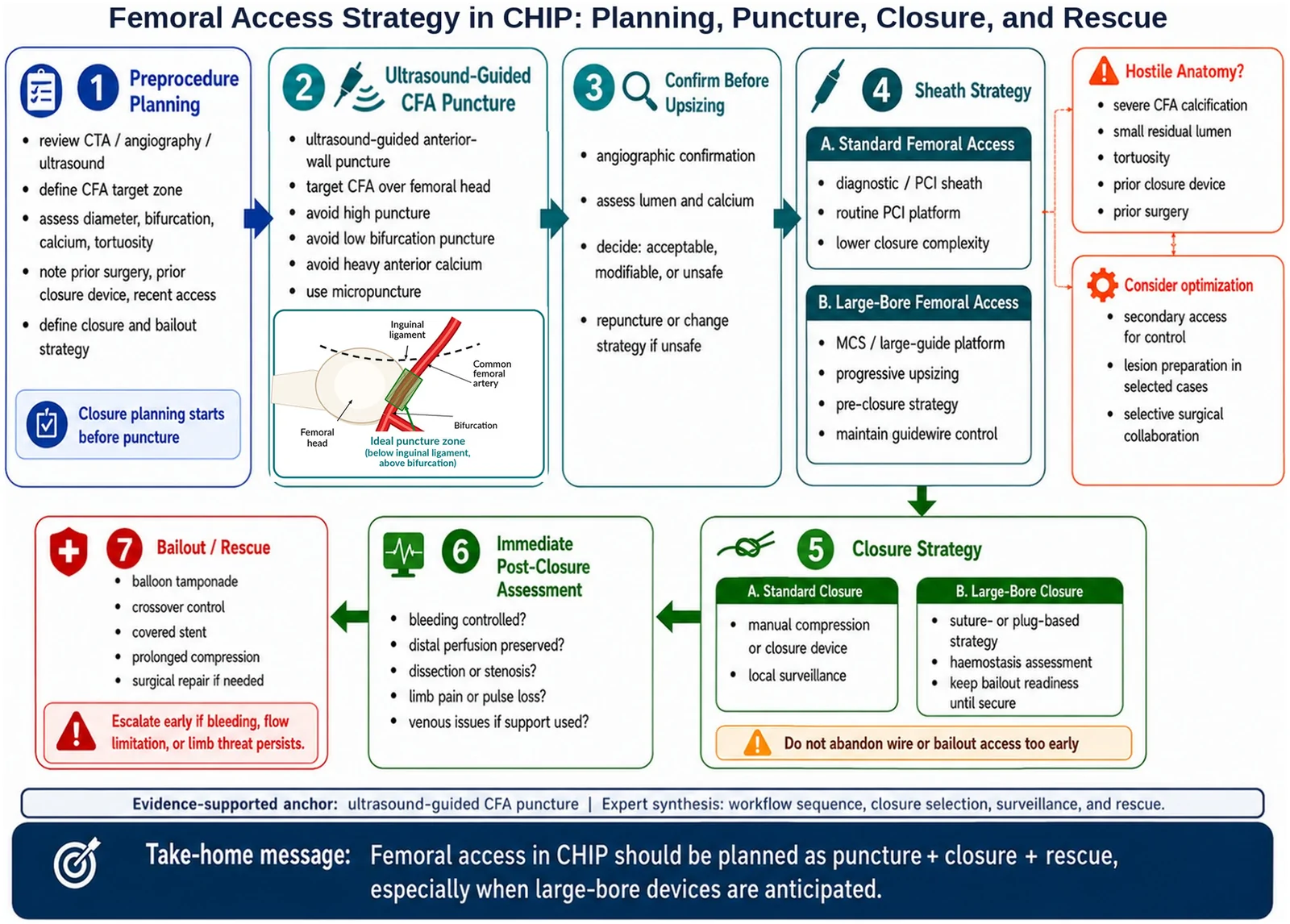
Femoral access strategy in CHIP: planning, puncture, closure, and rescue. Procedural workflow for standard and large-bore femoral access in CHIP. Safe femoral access requires preprocedural anatomical assessment, real-time ultrasound-guided common femoral artery puncture, confirmation before upsizing, selection of the sheath strategy, predefined closure planning, immediate post-closure assessment, and early bailout when necessary. Real-time ultrasound-guided anterior-wall CFA puncture is presented as the routine approach to optimise puncture precision and as the default before planned large-bore upsizing. Randomised data support higher first-pass success, and pooled coronary-trial data suggest fewer major bleeding/vascular complications; however, ultrasound guidance did not reduce the primary clinical endpoint in ULTRACOLOR, which tested ≥7 Fr complex-PCI access rather than MCS-sized access. (21–23, 73) Current ESC guidelines assign no separate recommendation class to ultrasound guidance. (8, 9) Evidence status: Ultrasound-guided CFA puncture is evidence-supported. The precise workflow sequence, confirmation and upsizing decisions, closure-device selection, surveillance, and bailout pathway are informed mainly by expert synthesis based on consensus and observational large-bore data (11, 12, 15, 26, 91); hostile-anatomy modifications are supported only by limited observational or feasibility data. (13, 16, 92) They have not been validated as a CHIP-specific algorithm. CFA, common femoral artery; CHIP, complex high-risk indicated percutaneous coronary intervention; CTA, computed tomography angiography; Fr, French; MCS, mechanical circulatory support; PCI, percutaneous coronary intervention.

Standard femoral access should be approached as a deliberate contemporary strategy rather than as a fallback after radial failure. Its principal strengths in complex PCI are stronger passive guide support, easier upsizing, broader device compatibility, and procedural simplicity when prolonged instrumentation or dual-catheter work is anticipated (7, 33). These advantages are particularly relevant in left main intervention, complex bifurcation PCI, some CTO procedures, and cases in which rapid escalation to large-bore support might become necessary (2, 7, 33).

However, the benefits of femoral access depend heavily on puncture quality. Real-time ultrasound has become central to this step. In the FAUST randomised trial, which enrolled 1,004 patients undergoing retrograde femoral arterial access, ultrasound guidance did not significantly improve overall common femoral artery cannulation compared with fluoroscopic guidance alone (86.4% vs. 83.3%; *p* = 0.17), but it did improve cannulation in patients with high femoral bifurcations (82.6% vs. 69.8%; *p* < 0.01), increased first-pass success (83% vs. 46%; *p* < 0.0001), reduced the number of attempts (1.3 vs. 3.0; *p* < 0.0001), reduced venipuncture (2.4% vs. 15.8%; *p* < 0.0001), shortened median time to access (136 vs. 148 s; *p* = 0.003), and lowered vascular complications at 30 days (1.4% vs. 3.4%; *p* = 0.04) (23). These data support ultrasound-guided puncture as a standard component of femoral planning in CHIP; pooled randomised evidence suggests particular benefit when a vascular closure device is used (21, 23). The coronary-specific ULTRACOLOR trial adds nuance for complex PCI requiring large-bore transfemoral access: among 544 patients randomised to ultrasound- vs. fluoroscopy-guided puncture for ≥7 Fr femoral complex PCI, ultrasound did not significantly reduce the composite of access-site BARC 2, 3 or 5 bleeding or vascular complications requiring intervention during hospitalisation (15.7% vs. 18.9%; *p* = 0.32), but improved first-pass puncture (91.8% vs. 85.2%; *p* = 0.02) (73). Taken together, these findings support routine use of real-time ultrasound for common femoral artery puncture to improve puncture precision, including before planned large-bore access, while recognising that ULTRACOLOR did not demonstrate a significant reduction in in-hospital bleeding or vascular complications (21, 23, 73).

Accordingly, standard femoral access in CHIP should be framed around three principles: common femoral rather than superficial femoral or external iliac puncture; real-time ultrasound-guided anterior-wall puncture as the default technique, with fluoroscopic or angiographic confirmation when appropriate (7, 21–23); and retention of immediate angiographic or endovascular control if puncture proves suboptimal or closure becomes problematic (12, 15). Even when sheath size is modest, this mindset aims to reduce the chance that a technically successful PCI is followed by a preventable access-site complication.

Hostile iliofemoral anatomy may also limit standard femoral or hybrid PCI when MCS is not planned. Severe common femoral calcification, a small residual lumen, iliac tortuosity, prior surgery, and diffuse peripheral artery disease can compromise puncture, closure, and bailout (12, 13, 15, 16). When no reliable puncture and closure zone exists, a technically adequate forearm or alternative-access strategy should be preferred; large-bore optimisation and alternative support access are addressed in Part II (7, 16).

Figure 3 summarises the forearm-first, femoral-first, and modified access pathways.

**Figure 3:**
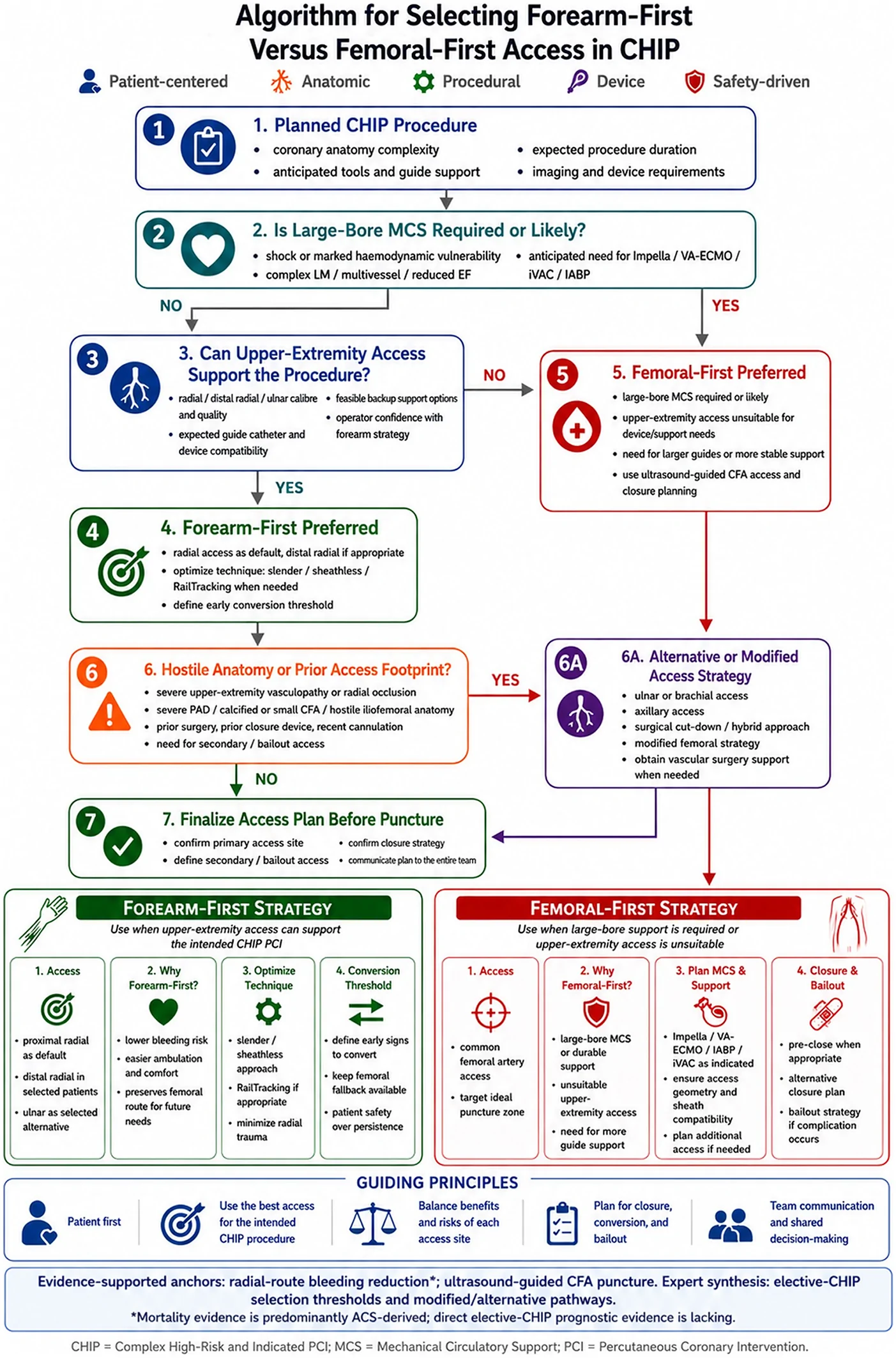
Algorithm for selecting forearm-first versus femoral-first access in CHIP. Practical algorithm for selecting forearm-first, femoral-first, or alternative access strategies in CHIP. The decision should integrate anticipated need for large-bore support, procedural feasibility from the upper extremity, guide-catheter support and lumen requirements, lesion subset, arterial anatomy, prior access footprint, bleeding risk, and operator experience. Access choice should follow the complete procedural plan and represent the smallest safe architecture that can deliver it and permit timely escalation. Evidence status: The overall decision tree, thresholds, and alternative-access pathways collectively constitute an author-derived expert synthesis and have not been prospectively validated. Randomised evidence supports radial-route bleeding reduction (27, 31), and ESC guidelines recommend radial access for coronary procedures (8, 9); however, mortality evidence is predominantly ACS-derived and direct elective-CHIP prognostic evidence is lacking. (31, 32) Ultrasound-guided femoral puncture is evidence-supported (21–23, 73); distal radial, ulnar, sheathless/RailTracking, axillary, and modified large-bore pathways rely mainly on observational or feasibility evidence and experienced-centre practice. Alternative and modified large-bore pathways require established endovascular and vascular-surgical rescue capability. ACS, acute coronary syndrome; CFA, common femoral artery; CHIP, complex high-risk indicated percutaneous coronary intervention; CTO, chronic total occlusion; EF, ejection fraction; IABP, intra-aortic balloon pump; LM, left main; MCS, mechanical circulatory support; PAD, peripheral artery disease; PCI, percutaneous coronary intervention; VA-ECMO, veno-arterial extracorporeal membrane oxygenation.

## Procedure-specific modifiers of access choice

### CTO and prior CABG

Contemporary PROGRESS-CTO data support radial-only access in selected CTO PCI with fewer access complications and similar adjusted technical success (74). Radial-only access was used in approximately one quarter of procedures; cases were less anatomically complex, underlining the importance of selection. Prior CABG and graft anatomy require individualised planning that balances support needs with conduit preservation. Prior-CABG CTO PCI has lower technical and procedural success and more challenging anatomy than CABG-naive PCI (75).

### Severe calcification and left-main/bifurcation PCI

Severe calcification and atherectomy are access-sensitive because larger burrs, intravascular imaging, and sequential calcium-modification tools increase guide and lumen requirements. In a large UK observational registry, radial access for rotational atherectomy was associated with lower major bleeding and access-site complications, with similar adjusted 30-day mortality and procedural success, when the intended equipment could be delivered (72). Sheathless and RailTracking strategies may preserve forearm feasibility in expert hands (34, 35, 71). Access should nevertheless be chosen for the complete lesion-preparation plan, not only for the first device (17).

Left-main and complex bifurcation PCI do not mandate femoral access, but they lower the threshold for a more supportive platform when prolonged wiring, two-stent techniques, kissing balloons, or extensive imaging are anticipated (18).

### CKD, obesity, and peripheral artery disease

In chronic kidney disease and ultra-low-contrast PCI, the preferred route is the one that supports a stable imaging-guided workflow and procedural completeness, rather than simply the smallest sheath (76). Obesity and peripheral artery disease require imaging-guided individualisation: upper-extremity access may avoid a difficult groin in obesity, whereas diffuse PAD may compromise both device delivery and closure (7, 12, 15). Moving proximally to brachial access can introduce a different complication burden rather than eliminate risk (60, 61).

Supplementary Appendix 1 and Table S1 provide detailed scenario-specific considerations.

## Management of forearm and standard femoral access complications

Complication management should distinguish mechanism. Radial spasm is treated with analgesia and sedation, intra-arterial vasodilators, forearm warming, and reactive hyperaemia (30). A limited, non-expanding forearm haematoma requires prompt compression at the bleeding source, elevation of the arm above heart level, and serial neurovascular assessment (30, 77). Antithrombotic interruption or adjustment should not be automatic; it should be individualised for clinically significant or expanding bleeding after balancing bleeding severity and procedural circumstances against thrombotic risk (77). For radial-artery perforation controlled by guide-catheter and/or cuff tamponade, protamine is typically not required; persistent or severe bleeding should prompt mechanism-specific escalation (78). Progressive swelling, severe pain, paraesthesia, or motor deficit should trigger urgent evaluation for perforation or compartment syndrome (30, 77).

Forearm prevention is based on artery preservation: the smallest effective outer profile, adequate anticoagulation, avoidance of repeated traumatic puncture, and haemostasis that preserves antegrade flow (28, 30, 36, 79). Radial artery occlusion may be clinically silent but can compromise future coronary, surgical, or dialysis access (28, 30, 79). Early imaging is appropriate when pulse loss, persistent pain, or neurological symptoms occur (28, 30).

Standard femoral complications require early anatomical clarification. Persistent bleeding, flow-limiting dissection, pseudoaneurysm, or threatened limb perfusion may require compression, crossover balloon control, covered stenting, or surgery; rescue is facilitated when a secondary route and guidewire control have been preserved (15, 26).

Supplementary Appendix 2 and Table S2 provide a structured prevention and bailout framework.

## Part II. Chip with planned MCS: integrated support-access architecture

### Selecting patients for planned support

Routine prophylactic support is not justified for every CHIP procedure. PROTECT II was a randomised device-comparison trial—Impella 2.5 vs. IABP, not support vs. no support—and was stopped early for likely futility; it showed stronger haemodynamic support but no significant reduction in the 30-day primary endpoint (80). CHIP-BCIS3 more directly compared planned Impella CP unloading with standard care and found no benefit in its hierarchical primary outcome, with no clear between-group differences in bleeding or vascular complications (81). By contrast, PROTECT I established feasibility only, and claims-based comparisons are observational and hypothesis-generating because device selection and residual confounding cannot be fully addressed (82, 83). Together with the neutral short-term primary outcome of BCIS-1, current randomised evidence does not support routine prophylactic MCS during nonemergent CHIP (80, 81, 84, 85). This is consistent with the 2024 ESC CCS recommendation that a microaxial flow pump may be considered in selected patients with heart failure with reduced ejection fraction undergoing high-risk PCI for complex coronary disease in experienced centres (Class IIb, Level C) (9). EAPCI consensus likewise states that MCS should not be used as the default strategy (1).

Patient selection and access planning are inseparable: a technically suitable access route is necessary but not sufficient. Anticipated haemodynamic benefit, myocardium at risk, ventricular function, lesion complexity, and procedural ischaemic burden should first be balanced against frailty, comorbidity, and alternative treatment options; the selected device must then be assessed against arterial and venous anatomy, total puncture burden, removal, closure, limb-perfusion, and rescue feasibility (1, 2, 10, 12). In CTO PCI, available evidence is observational: registry patients receiving elective MCS are more complex and experience more bleeding and in-hospital events (86), while small Impella-supported cohorts establish feasibility only and remain hypothesis-generating (87).

### Integrating coronary access and support-device access

With some exceptions, once MCS enters the procedural plan vascular access is no longer a single-site decision. The plan must integrate coronary access, support-device access, device monitoring and removal, closure, and bailout (2, 7, 10, 12). Single-access Impella techniques may reduce the number of punctures in selected cases, but the evidence is limited to small feasibility series (19). PCI through a VA-ECMO arterial return cannula has also been described in an isolated case report (88). Neither approach removes the need for an integrated rescue plan.

When support is selected, coronary and support access must be considered together. A radial or distal-radial PCI route combined with femoral support can preserve the femoral artery for the large-bore device, whereas a femoral PCI route may simplify device delivery when larger guides or extensive atherectomy are expected. The SHiP technique provides a carefully selected exception: a PCI sheath is advanced through the Impella CP haemostasis valve, allowing support and PCI through one arterial entry site. In the initial 17-patient series, valve bleeding was not observed and one vascular complication occurred (19). By design, however, relying on a single arterial entry may leave no independent route for angiographic control or bailout unless another access is obtained (12, 19).

Figure 4 details the distinct access architecture required when MCS is planned.

**Figure 4:**
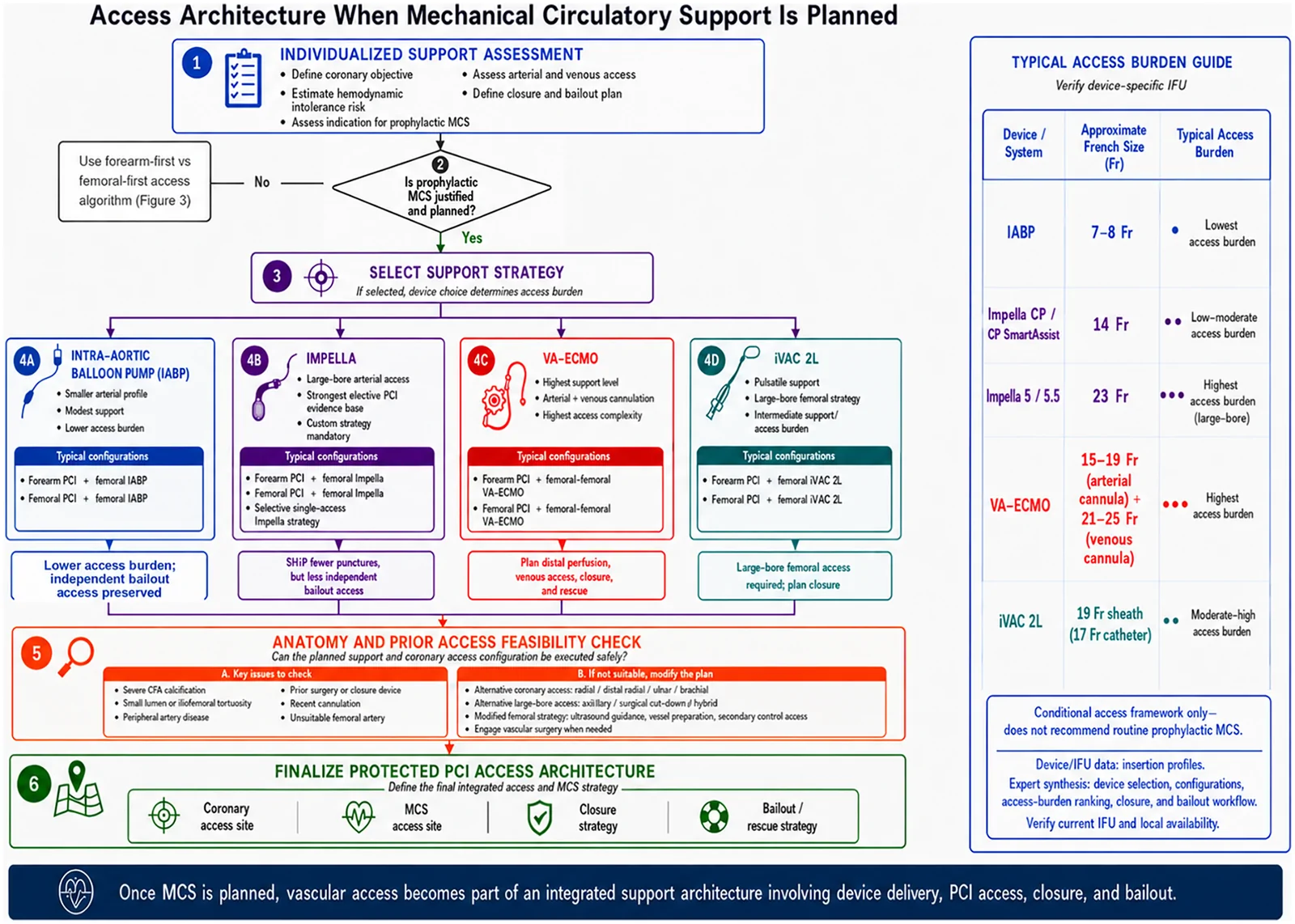
Access architecture when mechanical circulatory support is planned. Figure 4 applies only after individualised assessment of the clinical indication for prophylactic MCS; randomised evidence has not demonstrated a consistent routine prognostic benefit, despite the long-term mortality signal in BCIS-1. (80, 81, 84, 85) Access feasibility should inform, but not create, the indication for support. Once support is selected, access planning should integrate the device, coronary procedural requirements, arterial and venous anatomy, closure strategy, and bailout options. Hybrid strategies, such as upper-extremity PCI with femoral support access, may preserve coronary procedural feasibility while limiting additional access-site burden. Device-specific planning should account for the distinct vascular implications of intra-aortic balloon pump, Impella, veno-arterial extracorporeal membrane oxygenation, and iVAC 2L support. Approximate device profiles are drawn from device-specific literature and consensus for IABP and Impella (2, 10, 12, 14), VA-ECMO, (96–101, 105) and iVAC 2L (102–104); current device-specific instructions for use should govern. Alternative and modified large-bore pathways are experienced-centre strategies requiring established endovascular and vascular-surgical rescue capability. (13, 16, 90–92) Evidence status: This is an author-derived, conditional access-planning framework rather than an MCS-indication algorithm. Device selection, access configurations, comparative burden ranking, anatomy modification, closure, and bailout pathways are informed mainly by expert synthesis based on observational evidence and consensus. The figure should not be interpreted as recommending routine prophylactic MCS or as implying a proven prognostic benefit for any support device. CFA, common femoral artery; CHIP, complex high-risk indicated percutaneous coronary intervention; Fr, French; IABP, intra-aortic balloon pump; IFU, instructions for use; MCS, mechanical circulatory support; PCI, percutaneous coronary intervention; SHiP, single-access for Hi-risk PCI; VA-ECMO, veno-arterial extracorporeal membrane oxygenation. French sizes are approximate and should be verified against current device-specific instructions for use.

## Large-bore femoral access and closure

Once MCS or another large-calibre device is planned, large-bore femoral access should be treated as an integrated puncture–closure–rescue workflow because puncture accuracy, vessel size and calcification, sheath-to-artery compatibility, and closure feasibility jointly determine vascular risk (2, 7, 11, 12, 15). Comparative closure evidence is derived mainly from TAVR, not CHIP. In the pilot randomised MASH trial, MANTA did not demonstrate superiority to double ProGlide for access-site vascular complications or clinically relevant bleeding. Modified closure-device failure was less frequent with MANTA, but bailout differed, with additional closure devices used more often after ProGlide and numerically more covered stents or surgical rescue after MANTA (11). An older retrospective TAVR comparison of Prostar and ProGlide likewise showed device-dependent performance but cannot define a contemporary preferred strategy (89). These data do not identify a universally superior closure device for large-bore CHIP.

Strategy should therefore be individualised to femoral anatomy, access profile, operator experience, and the anticipated rescue pathway. Planning includes assessment of vessel size and calcification (7, 12, 15). Ultrasound-guided common femoral entry should routinely be used to optimise puncture precision (21–23, 73). Micropuncture, progressive upsizing, pre-closure when appropriate, and retention of guidewire or crossover control should continue until haemostasis and distal flow are confirmed (12, 15). Anterior-wall calcium may impair suture anchoring, whereas plug failure may reflect depth error, small vessel diameter, high puncture, or malapposition (15). Balloon tamponade, covered stenting, and surgical conversion must be immediately feasible when closure fails (11, 15, 26). Detailed prevention and escalation steps are provided in Supplementary Appendix 2 and Table S2.

### Hostile iliofemoral anatomy: optimisation and alternative support access

Hostile iliofemoral anatomy is one of the most important reasons why MCS access cannot be reduced to a routine femoral default. Severe common femoral calcification, small residual lumen, eccentric or circumferential plaque, iliac tortuosity, prior surgery, and diffuse peripheral artery disease affect device delivery, closure feasibility, and bailout success (12, 13, 15, 16).

Evidence for modified femoral, axillary, and other alternative large-bore strategies consists mainly of case reports, small series, feasibility registries, and expert-derived practical guidance; no randomised CHIP comparison defines a preferred approach (13, 16, 90–92).

Targeted angioplasty of a heavily calcified femoral puncture or closure zone, as described with the FEM-PREP technique, may facilitate suture-mediated closure in selected anatomies; however, the evidence is limited to a small case series and prospective validation is required (13). A related proof-of-concept is intravascular lithotripsy-assisted delivery across calcified iliofemoral disease, which should remain a highly selected expert-centre strategy rather than a routine substitute for a safe access route (90).

The growing sophistication of endovascular bailout should not obscure the continuing role of selective surgical cutdown and surgical collaboration. Selective surgical exposure may be considered from the outset when there is extensive common femoral or iliofemoral disease, severe calcific obstruction, no reliable percutaneous closure zone, or when large-bore complications are unlikely to be controlled durably by endovascular means alone; in such cases, direct arterial visualisation and immediate repair capacity are part of the access strategy rather than evidence of percutaneous failure (12, 15, 16). Covered-stent bailout can be effective when it is anticipated and technically supported, but it should not replace careful access selection (26).

Axillary Impella access can be considered when iliofemoral anatomy is unsuitable. Small coronary series and the prospective ARMS registry demonstrate high implantation success, but bleeding, brachial-plexus symptoms, closure, stent-graft use, and surgical explant remain relevant (91, 92).

In ARMS, percutaneous axillary implantation succeeded in 98% of 102 patients. Device removal nevertheless required a spectrum of strategies, including closure devices, balloon assistance, stent grafts, or planned surgical explant, and bleeding or brachial-plexus symptoms still occurred (92). Axillary access is therefore supported as a feasibility option for hostile iliofemoral anatomy in experienced centres with planned closure and rescue capability, not a proven low-complexity alternative to femoral access (91, 92).

## Device-specific access considerations

### Impella

Impella ECP uses a 9 Fr insertion and removal profile, but mature comparative data on clinical outcomes, vascular complications, and closure remain limited (2, 93). Separately, the investigational VITALYST system was evaluated in a 15-patient early-feasibility study using a purpose-built 14 Fr insertion sheath with an integrated side port for PCI catheters up to 8 Fr (94).

The arterial price of Impella is measurable. In a 61-patient high-risk PCI cohort using standardised femoral access, major vascular complications occurred in 5 patients (8%); peripheral arterial disease was associated with these events in regression analysis (14). The finding supports careful vessel selection, image-guided puncture, and a predefined closure and rescue strategy rather than treating access risk as an unavoidable property of the pump.

In an observational PROTECT III analysis of 1,237 Impella-supported patients, 493 (39.9%) were aged at least 75 years; compared with younger patients, they underwent more left-main PCI, atherectomy, and femoral coronary access. In-hospital vascular complications were similar, but one-year mortality was higher despite comparable short-term major adverse events (95). Large-bore access outcomes therefore need to be interpreted within frailty, comorbidity, and prognosis rather than in isolation.

### VA-ECMO

VA-ECMO provides the most comprehensive cardiopulmonary support but requires the most complex access architecture (2, 10, 12). It usually requires large-bore arterial and venous cannulation, limb-perfusion planning, decannulation, and two-site haemostasis (10, 12). Elective series show high procedural success, but reported complications have included surgical femoral repair (96, 97), limb ischaemia, haematoma, and transfusion (97), dialysis (97, 98), and femoral-vein thrombosis (99). A completely percutaneous local-anaesthesia workflow was feasible in an expert centre, with access complications still occurring in 17.1% (100). A small single-centre randomised trial suggested fewer intraprocedural life-threatening complications with prophylactic VA-ECMO in highly selected complex PCI, but admission was longer and invasive-procedure complications included bleeding, lower-extremity venous thrombosis, and femoral arterial thrombosis with limb ischaemia (101).

The individual series illustrate why protocolisation matters. In a Dutch two-centre cohort, PCI succeeded in all 14 patients and 93% survived to discharge, with ECMO-related complications in 14%, including a femoral-vein thrombus (99). Other elective series reported high procedural success but included femoral repair, limb ischaemia, transfusion, and dialysis (96–98). These data support superselected use in centres able to plan arterial and venous access, distal perfusion, decannulation, and endovascular or surgical rescue before cannulation.

### iVAC 2L

PulseCath iVAC 2L provides pulsatile transaortic support but remains a large-bore femoral strategy. Pilot, early German, and later registry evidence is observational and non-comparative; access-event rates should not be compared directly because definitions and adjudication differed (102–104).

In the first iVAC 2L pilot, implantation succeeded in 13 of 14 patients and one major procedural complication was related to the 19 Fr access sheath (102). The first German experience achieved device placement in 17 of 20 patients and PCI success in all patients, but included severe femoral bleeding and a false aneurysm requiring surgery (103). The international registry reported major vascular complications in 2.1% and major bleeding in 1.0%, which is reassuring but still places iVAC 2L within a large-bore femoral workflow (104).

### IABP and comparative protected-PCI evidence

IABP has the smallest arterial profile but the least haemodynamic support (2, 12). In BCIS-1, elective IABP did not reduce the short-term primary composite endpoint; the later mortality signal was insufficient to establish a routine prognostic benefit (84, 85).

In a multicentre retrospective observational comparison of prophylactic Impella CP and VA-ECMO in 41 CHIP procedures, periprocedural haemodynamic instability, major adverse events, and 30-day mortality were not significantly different. Bleeding was numerically more frequent with Impella and was often attributed to the additional non-Impella access, illustrating that total access burden may be more important than the support route alone (105).

Table 2 summarises device-specific access implications. Device-specific French sizes are approximate and should be verified against the current instructions for use.

**Table 2:** Access implications of planned mechanical circulatory support in CHIP.

Support strategy	Typical access configuration	Evidence-informed practical role	Reported vascular/bleeding profile^a^	Practical closure/bailout strategy^b^
IABP	Femoral artery, usually 7–8 Fr; may accompany radial PCI. (2, 12)	Lowest access burden but modest support; routine benefit unproven. (2, 12, 84, 85)	BCIS-1 elective-IABP arm: 3.3% access-site complications; 3.3% major bleeding; 19.2% major/minor bleeding under older definitions. (84)	Manual compression or selected 7–8 Fr closure device; no routine large-bore preclosure. (12, 84)
Impella (standard dual-access)	Usually 14 Fr femoral support plus separate PCI access, often radial; larger platforms need device-specific planning. (10, 12, 14, 80–83, 95)	Established platform; routine prophylactic benefit unproven. Hybrid radial–femoral use may limit burden. (10, 12, 80, 81)	CHIP-BCIS3 pump arm: major vascular 1.4%, major bleeding 10.8%, minor vascular 15.5%; an observational cohort reported 8% major vascular complications. (14, 81)	Anatomy-guided double-suture preclosure or selected plug; close the PCI site separately and preserve wire/crossover control until haemostasis and distal flow. (12, 14)
Impella ECP	9 Fr insertion/removal profile with an expandable deployed profile; currently an early or investigational option. (2, 93)	Potential lower-entry-profile support; comparative outcomes unavailable. (2, 93)	Not estimable; mature comparative vascular, bleeding, and closure data are unavailable. (2, 93)	Follow current device-specific IFU/study protocol; no evidence-based preferred closure strategy. Maintain bailout capability. (2, 93)
Impella with SHiP	Single 14 Fr Impella CP femoral access used for both support and PCI. (19)	Avoids a second arterial puncture; feasibility evidence is limited. (19)	Not estimable. In a 17-patient series, no bleeding occurred through the Impella-sheath haemostasis valve during the procedure or after PCI-sheath removal; one patient had an iliac thrombus/vascular complication of uncertain attribution. (19)	Use anatomy-selected suture or plug closure; plan independent bailout access because the second puncture is avoided. (12, 19)
VA-ECMO	Femoral arterial plus venous cannulation; distal perfusion may require another access. (96–101, 105)	Most comprehensive support, but highest total access complexity. (96–101, 105)	A 35-patient expert-centre registry reported 17.1% access complications, all minor; bleeding/transfusion estimates vary across small cohorts. (100)	Plan decannulation before cannulation: percutaneous closure when suitable, otherwise surgical repair; define venous haemostasis, limb surveillance, and immediate rescue. (12, 96, 97, 99–101)
PulseCath iVAC 2L	17 Fr catheter delivered through a 19 Fr expandable femoral sheath. (102–104)	Pulsatile support that remains a large-bore femoral strategy. (102–104)	Registry (*n* = 293): major vascular 2.1%, major bleeding 1.0%; definitions/adjudication were nonuniform. (104)	The initial feasibility study used ultrasound-guided CFA access; both feasibility studies used double-suture preclosure. Retain wire/crossover and surgical bailout readiness. (102, 103)

ECMO, extracorporeal membrane oxygenation; IABP, intra-aortic balloon pump; IFU, instructions for use; MCS, mechanical circulatory support; SHiP, Single-access for Hi-risk PCI.

a Rates are study-specific and not directly comparable because populations, devices, access configurations, definitions, adjudication, and follow-up differ; bleeding is not always access-site-specific.

b Closure strategies derive mainly from observational evidence, consensus, and IFU rather than head-to-head CHIP trials; individualise to anatomy, device, anticoagulation, and centre expertise. This table describes access after support selection and is not an indication for routine prophylactic MCS. French sizes are approximate; verify current IFU.

## Urgent or bailout support

The difference between planned and bailout support is clinically important. In an observational analysis of 1,028 Impella-supported nonemergent high-risk PCI procedures, mortality was markedly higher when Impella was inserted as bailout after haemodynamic collapse than when support was planned (106). This does not prove benefit from routine support, because bailout patients were already deteriorating, but it emphasises the need to decide before PCI whether rescue support is realistically deployable.

In cardiogenic shock or rapid deterioration, speed and support capability may outweigh some long-term access advantages. The preferred route is the fastest safe option that preserves a realistic rescue pathway (2, 10, 12). The 2023 ESC ACS guideline permits short-term MCS in severe or refractory cardiogenic shock (Class IIb, Level C) and advises against routine IABP in ACS-related shock without mechanical complications (Class III, Level B); these acute recommendations should not be extrapolated to elective CHIP (8). The bleeding advantage of radial coronary access is established by randomised evidence in ACS (31), whereas cardiogenic-shock comparisons remain observational and susceptible to selection bias (30). Radial access may preserve femoral routes for MCS but should not delay revascularisation or circulatory support (30). When Impella is used, single-access or alternative-access concepts described in selected patients may help (19, 91, 92), but should not distract from rapid haemodynamic support and early control of bleeding or limb perfusion.

## Large-bore and MCS complication prevention and bailout

Persistent bleeding, flow-limiting dissection or stenosis, failed closure, or threatened limb perfusion requires prompt anatomical definition and escalation to balloon tamponade or crossover control, covered stenting, or surgery; rescue is more feasible when secondary access and guidewire control have been preserved (15, 26). Small TAVR covered-stent series support bailout feasibility in selected patients, not comparative superiority or long-term durability (26).

MCS-related risk remains device-specific: Impella may cause large-bore arterial injury and bleeding (14), VA-ECMO adds venous thrombosis, decannulation, and limb-perfusion risk (96–101), and iVAC 2L remains a large-bore femoral strategy despite favourable registry signals (102–104). Prevention begins before insertion with vessel assessment, ultrasound-guided puncture, a defined closure plan, and early consideration of crossover control or distal perfusion (12, 15, 26).

Supplementary Appendix 2 and Table S2 provide detailed prevention, surveillance, and escalation pathways.

## Discussion

### Access within comprehensive CHIP planning

Vascular access is an enabling component of CHIP planning, not an independent determinant of procedural success. The team should first define the revascularisation objective and clinical indication (3, 4, 9). Lesion preparation, intravascular imaging, and calcium-modification requirements should be specified before access selection (1, 17, 18). Haemodynamic tolerance, potential need for MCS, and available operator and institutional expertise should be assessed separately (1, 2, 80, 81). The access architecture should then provide the required guide support and device compatibility with the lowest reasonable vascular burden and a credible rescue pathway (1, 7, 12, 15).

Within this broader plan, separating CHIP without planned MCS from MCS-supported CHIP remains useful because their access consequences differ. Forearm access should remain central when it can support the complete intervention; femoral or hybrid access should be selected early when guide, device, or support requirements exceed a safe forearm platform. When MCS is planned, coronary access, support access, device removal, closure, and bailout should be designed together. An explicit conversion threshold avoids both persistence with an inadequate forearm platform and unnecessary default to femoral or large-bore access.

### Implications of the two-pathway framework

In CHIP without planned MCS, forearm-first access can reduce bleeding and preserve one or both femoral arteries for emergency support, angiographic control, or endovascular rescue (7, 12, 30). That advantage is lost when the upper-extremity route cannot provide adequate guide support or device compatibility. A declared conversion threshold is therefore part of a forearm-first strategy, not evidence that it has failed. Hybrid radial–femoral or dual-forearm approaches may be preferable when contralateral injection, graft engagement, or additional support is anticipated (18, 33, 63, 67).

In supported CHIP, access anatomy should inform but cannot create the support indication. Expected haemodynamic benefit, coronary objective, total puncture burden, device removal, closure, distal perfusion, and rescue capacity should be assessed together (1, 2, 10, 12). The same support device can carry different vascular risk according to whether PCI is radial or femoral, access is single or dual, and venous or distal-perfusion cannulation is required (10, 12, 14, 19, 105). Recent randomised trials have not established a consistent clinical benefit from routine planned MCS in elective high-risk PCI (80, 81, 84, 85).

### Operator and institutional competence

Operator and institutional experience are components of procedural safety rather than generic modifiers of access selection. Distal radial, ulnar, brachial, sheathless/RailTracking, axillary, large-bore closure, and VA-ECMO strategies have distinct learning curves and bailout requirements. Outcomes reported from expert centres may reflect patient selection, procedural volume, standardised protocols, imaging proficiency, and immediate access to endovascular or surgical rescue; they should not be assumed reproducible where these competencies are absent. EAPCI consensus therefore emphasises institutional protocols and competence in radial and ≥10 Fr femoral access, closure, imaging, complex PCI, MCS, and complication rescue (1).

In the observational Polish National PCI Registry (ORPKI) analysis of 539 operators at 151 centres, mean operator-level mortality for femoral PCI increased across strata of radial utilisation from 0.26% among operators using radial access in <25% of procedures to 1.41% among those using it in >75% (*p* = 0.003); femoral access-site bleeding did not differ significantly across strata (*p* = 0.30). These unadjusted associations cannot establish that radial specialisation causes poorer femoral outcomes: access was operator selected, radial utilisation was an imperfect proxy for proficiency, and lesion complexity, sheath size, closure devices, lifetime experience, and post-discharge outcomes were unavailable (107).

The practical implication is not to reverse radial-first practice but to maintain and audit femoral and large-bore competence, define early conversion thresholds, and confine brachial, axillary, transcaval, and modified large-bore approaches to trained teams in experienced centres with protocolised imaging and closure and immediate endovascular and vascular-surgical rescue capability (1, 16, 60–62, 91, 92).

### Current evidence gaps and future directions

No randomised study has established radial-first as optimal for every elective CHIP procedure or identified a subgroup with superior clinical outcomes from routine femoral-first PCI (27, 31, 32). The mortality signal for radial access derives chiefly from ACS populations; direct prognostic evidence in elective CHIP is lacking (31, 32). Access should therefore follow the procedural mission—guide-lumen and support requirements, dual injection, lesion modification, anticipated duration, and rescue needs—modified by bleeding risk, arterial anatomy, and expertise (1, 7, 17, 18, 33). Femoral-first coronary access is most defensible when an upper-extremity platform cannot safely deliver the required equipment or support. Large-bore MCS requires an integrated femoral or alternative support route, but not necessarily femoral coronary access; a hybrid radial–femoral architecture may remain appropriate (2, 10, 12).

CTO comparisons are predominantly observational or single-centre, with less complex anatomy often selected for radial access (63–67, 74). Evidence remains limited in prior-CABG CTO, retrograde procedures, dual injection, and large-guide strategies (33, 74, 75). Future studies should jointly report procedural success, crossover, contrast, equipment delivery, and access complications.

Neurological risk also requires cautious interpretation. In the ORPKI registry of 81,587 acute myocardial infarction PCI procedures, only 17 periprocedural strokes occurred. Radial access itself was not an independent predictor; chronic kidney disease and a lower proportion of each operator's PCIs performed radially were independently associated with stroke, while crossover was more frequent among stroke cases. The low event count, local diagnosis, absence of stroke typing, less-than-24 h ascertainment, and acute myocardial infarction setting make these findings exploratory and not directly generalisable to elective CHIP. They support further study of catheter manipulation, avoidable crossover, and route-specific expertise rather than abandonment of radial access (108).

Future research should compare access strategies prospectively in complexity-stratified elective CHIP. Core datasets should capture the complete access architecture—coronary and support routes, guide profile, crossover, closure, and rescue—together with neurological events, patient-reported outcomes, and operator- and centre-level learning curves. DISCO COMPLEX, ULNART, and PROTECT IV will address selected gaps, but adequately powered pragmatic trials and validated decision tools remain needed (55, 59, 109).

Future access programmes should treat radial preservation as a bundle—atraumatic puncture, the smallest feasible outer profile, adequate anticoagulation, vasodilator use when appropriate, patent low-pressure haemostasis, short compression, and postprocedural patency assessment—rather than as distal radial access alone (28, 30). The 0.91% forearm radial-artery-occlusion rate observed after conventional transradial access with optimised contemporary practice in DISCO RADIAL tempers the absolute incremental benefit of routine dTRA (46). Ultrasound may mitigate dTRA's puncture penalty, but completed evidence remains principally procedural and centre- and operator-dependent (110). ALPHA&OMEGA (NCT06835829) is a registered, open-label, dual-randomised pragmatic trial (estimated *n* = 480) comparing conventional with distal radial access; within the planned 240 dTRA participants, a second 1:1 randomisation tests ultrasound-guided vs. conventional puncture. Its registered primary outcomes are haemostasis duration and successful sheath placement. ClinicalTrials.gov lists the study as active, not recruiting, with actual primary completion on 30 July 2026; results remain awaited and none are posted. It is not CHIP-specific and excludes ST-segment elevation myocardial infarction and cardiogenic shock (111).

Radial-first adoption must not erode femoral or large-bore competence because selected CHIP procedures still require femoral access for guide support, device compatibility, or MCS. Evidence for the proposed “radial paradox” is conflicting and observational. An adjusted Blue Cross Blue Shield of Michigan Cardiovascular Consortium (BMC2) analysis associated high radial use with more femoral vascular complications despite fewer vascular complications overall (112). Tokarek et al. reported higher femoral-PCI mortality across radial-use strata but only a non-significant bleeding trend (107). Large adjusted BCIS and National Cardiovascular Data Registry analyses did not identify clinically meaningful deterioration in femoral outcomes (113, 114). These findings support structured maintenance and audit of ultrasound-guided femoral, large-bore, closure, and bailout competence rather than retreat from radial-first practice.

Simulation may support structured acquisition and rehearsal of these infrequently used skills, particularly among coronary operators without regular structural-intervention or MCS exposure, but outcome evidence remains limited. A four-centre before–after study associated femoral-access simulation with fewer complications and earlier proficiency among first-year fellows (115). In a small randomised radial-puncture training study, the simulation group reported lower preprocedural stress without significant improvement in puncture success or complications (116). No study has established that simulation improves contemporary ultrasound-guided large-bore CHIP outcomes.

An artificial-intelligence-enabled robotic system for femoral-vein access has been evaluated only in phantoms and porcine models; use in human arterial access and CHIP remains investigational (117). Emerging closure technologies also require comparative CHIP validation; Frontier V was a small, single-arm TAVR/endovascular aneurysm-repair feasibility study, not a CHIP trial (118).

## Conclusions

Forearm access retains advantages concerning bleeding, mobilisation, comfort, and vessel preservation when procedural demands can be met from the upper extremity. Femoral access remains indispensable for maximal support, large-bore devices, and some equipment-intensive procedures. Within a comprehensive procedural plan, the preferred access architecture is the least invasive configuration that safely supports the intended revascularisation and timely rescue; the access plan is complete only when puncture, procedural requirements, closure, and bailout have been defined. Making those decisions explicit before puncture also improves team communication and clarifies when conversion or escalation should occur in routine clinical practice.
