# Supplementary material for: Vascular access options for complex high-risk indicated percutaneous coronary interventions: contemporary strategies, trade-offs, and practical algorithms
Source: Front Cardiovasc Med. 2026 Sep 28;13:1883827. doi: 10.3389/fcvm.2026.1883827 (PMC13655390; doi:10.3389/fcvm.2026.1883827)
Supplement: Supplementary file 1 [file Supplementaryfile1.docx]

# Supplementary Material

## Generative AI-assisted figure refinement documentation

The initial figure concepts and layouts were created by the authors using Microsoft PowerPoint. The figures were subsequently refined for graphical clarity and consistency using ChatGPT (version: ChatGPT web interface, accessed on 15 May 2026; model: GPT-5.5 Pro; source: OpenAI). The scripts used for figure refinement are provided in the Supplementary Material. The authors reviewed and verified all final figures to ensure that they accurately reflect the content of the manuscript and that no unsupported scientific content, data, references, or interpretations were introduced by the AI-assisted refinement process. The tool was ultimately used for language proofreading, consistency checks, and readability improvements of author-written text. The authors independently verified all scientific statements, numerical data, references, and final figures and take full responsibility for the submitted work. Further details are provided in the Supplementary Material.

Tool: ChatGPT by OpenAI (GPT-5.5 Pro; OpenAI, San Francisco, CA, USA).

Scope of use: editorial and visual refinement support for figures; no original data generation; no autonomous scientific interpretation; no authorship role.

## Prompt and output documentation

### Entry 1: Initial figure-refinement request

User prompt:

Hello. I wrote this review article for Frontiers in CV medicine. It's basically ready for submission but my Figures are quite basic currently. Could you generate some nice Figures optimals for a scientific journal (nice graphic with some icons/anatomical images whenever needed). Please check the journal requirements to make sure that whatever you generate will fit perfectly the journal's requirements. I will need them in TIFF format at 300dpi. Don't generate anything for now because first i would like to know whether this material i'm sharing with you is enough or not.

ChatGPT output:

I’ll first check the current Frontiers figure specifications, then compare them against the manuscript and figure blueprint you uploaded so I can tell you what is sufficient and what is still missing.

Yes — the material you shared is enough to move forward, especially because you provided both:

The full manuscript, with the scientific context and figure legends for Figures 1–6. The manuscript clearly defines the intended role of each figure: access-planning framework, upper-extremity options, femoral workflow, access-selection algorithm, MCS access architecture, and safe-access checklist.

The PowerPoint figure blueprint, which already contains the core text, box hierarchy, workflow logic, and arrow directions, including notes that some algorithmic figures should be more vertical.

For Frontiers in Cardiovascular Medicine, the key production requirements are also compatible with what you want: figures should be submitted individually, in numerical order, at 300 dpi at final size, with 1-column width = 85 mm or 2-column width = 180 mm, text no smaller than 8 pt, and lines no thinner than 2 pt. Frontiers accepts TIFF/JPEG, and figures must be in RGB color mode.

My recommendation would be to design these mostly as 2-column figures, especially Figures 3–6, because they are dense workflow/algorithm figures. At 300 dpi, a 2-column figure corresponds to roughly 2126 px wide; a 1-column figure is only about 1004 px wide, which would make the text too small for your current algorithms.

What is not strictly missing, but would improve the final result:

A decision on whether you want the anatomical elements to be clean schematic vector illustrations or more realistic anatomical-style drawings. For journal safety, I would avoid internet-derived anatomy unless licensing is explicit; original schematic anatomy/icons are safer because Frontiers requires permissions for re-used, adapted, or modified copyrighted material, and all published figures fall under a CC-BY license.

A final preference on visual style: “minimal scientific infographic,” “JACC-style central illustration,” or “more anatomical/clinical.” I can infer this, but your preference would help.

For Figure 5, the PowerPoint mentions a “reference image” for checking text/arrows/layout; if the non-editable reference image is visually important, it should remain available in the PPT during production.

So, in practical terms: yes, we can proceed with what you uploaded. I would generate each figure as an editable source master plus final RGB TIFF, 300 dpi, respecting Frontiers’ 180-mm width, page-height limit, minimum font size, line thickness, and arrow logic. The only thing I would avoid is using non-original anatomical images unless you provide licensed/permission-safe material.”

### Entry 2: Follow-up figure-refinement prompt

User prompt:

Ok. we can move forward them. Anyways when you generate images that shouldn't infringe copyright (you cannot just use other existing images published material). Also be careful to properly representing the different arrows in the each Figure draft i have created

### Entry 3: Follow-up figure-refinement prompt

User prompt:

Figure 1 is nice but i wonder if you could make those arrows more "dynamic

In figure 2 the image for the radial artery is incorrect (you point to the ulnar artery); also the figure for the distal radial artery is incorrect (you should show the artery in the anatomical snuffbox)

Figure 3 looks good

In Figure 4 maybe you could improve the graphic of the box "general principles"

In Figure 5 in the "typical access burden guide" possibly you should add the size in French for each system. Also you could eliminate the asterisk and the part it refers to "values are typical ranges...."

In Figure 6 maybe you can add some nice icons in each box”

### Entry 4: Follow-up figure-refinement prompt

User prompt:

In Figure 2 the image for the radial and distal radial artery are still wrong (both images indicate the ulnar artery).

### Entry 5: Follow-up figure-refinement prompt

User prompt:

Ok. The image for the distal radial artery is now good but not yet the one for the proximal radial (you still pointing to the ulnar). If it can helps the radial artery is the closest one to the thumb

### Entry 6: Follow-up figure-refinement prompt

User prompt:

not quite yet there. for the proximal radial artery you can use the same image you used for the ulnar but simply the sheath need to be on the other artery closest to the thumb

### Entry 7: Follow-up figure-refinement prompt

User prompt:

not perfect but it could go. now moving to Figure 5 the sheath needed for the IVAC 2L should be 18Fr while you didn't mention the French size for the Impella 5 and 5.5 versions that should be 23Fr

### Entry 8: Follow-up figure-refinement prompt

User prompt:

ok. now everything looks good. GREAT

ChatGPT output:

Great — glad we finally got the figures to the right level.

The next practical step would be to export the final approved versions as 300 dpi RGB TIFFs, with the correct Frontiers sizing, and ideally keep a separate editable/source version for any last-minute journal or reviewer requests.”

## Author verification statement

The authors confirm that all final figures and manuscript content were reviewed and verified by the authors. The authors take full responsibility for the accuracy, originality, and integrity of the final submitted material.
