# Supplementary material for: Vascular access options for complex high-risk indicated percutaneous coronary interventions: contemporary strategies, trade-offs, and practical algorithms
Source: Front Cardiovasc Med. 2026 Sep 28;13:1883827. doi: 10.3389/fcvm.2026.1883827 (PMC13655390; doi:10.3389/fcvm.2026.1883827)
Supplement: Supplementary file 2 [file Supplementaryfile2.docx]

# Supplementary Material

Citation numbers correspond to the revised main manuscript; references cited in this supplementary material are listed at the end of this file.

## Supplementary Figure S1. Upper-Extremity Access Options for Complex PCI and CHIP.

Comparative overview of upper-extremity access options in CHIP. Proximal radial access remains the default upper-extremity strategy in many cases because of its mature evidence base, low bleeding burden, and procedural familiarity.(28–31) Distal radial access may improve radial preservation and may shorten haemostasis but is associated with more difficult puncture and higher crossover.(42–47) Its use for selected complex PCI and CTO is supported mainly by observational experience.(48–51) Ulnar access is a selective alternative when radial access is unavailable or intentionally preserved.(28,56–58) Evidence regarding transulnar use in patients with ipsilateral RAO remains limited and hypothesis-generating.(59) Brachial access should remain a highly selected rescue route because of its higher complication burden.(60–62)

Abbreviations: CHIP, complex high-risk indicated percutaneous coronary intervention; CTO, chronic total occlusion; PCI, percutaneous coronary intervention; RAO, radial artery occlusion.

# Supplementary Appendix 1. Scenario-specific access considerations

The following sections retain detailed scenario-specific considerations that were condensed in the revised main manuscript.

## Chronic total occlusion PCI

CTO PCI illustrates how access strategy shapes technique. Dual injection, larger guides, contralateral visualization, and retrograde crossing often make access planning decisive.(33) In the BCIS registry of 26,807 CTO PCI cases, the radial–femoral comparison was restricted to 6,480 procedures performed in 2012–2013. In this subset, femoral access was used for more complex procedures and was associated with more access-site complications, periprocedural myocardial infarction, major bleeding, transfusion, and 30-day mortality. Procedural success was lower in the femoral cohort, reinforcing that radial–femoral outcome comparisons in CTO PCI are highly selection-sensitive.(63)

Contemporary data suggest that this success gap has narrowed. In a 3,790-case international CTO registry, radial-only, radial–femoral, and femoral-only access had similar technical and procedural success; major bleeding was lowest with radial-only access and highest with combined radial–femoral access.(64) A larger contemporary PROGRESS-CTO analysis of 10,954 cases extends this message: radial-only access was used in 24% of CTO PCIs and, after adjustment, was associated with fewer access-site complications with similar technical success and MACE compared with femoral access, although radial-only cases again had lower angiographic complexity.(74) A meta-analysis and the VA CART analysis also support comparable procedural success with selected radial CTO PCI, while access-site safety advantages are not uniform across populations and case mix.(65,66) These data support broader radial feasibility but not universal radial superiority, because radial cases were often less complex.

Randomised data reinforce this nuance. In FORT CTO, technical and procedural success were similar with radial and femoral access, while access-site complications were lower with radial access.(67) Crossover and support needs should nevertheless remain part of the procedural plan, especially in equipment-heavy CTO PCI.(33,67)

Distal radial and alternative forearm strategies further broaden the CTO toolkit. Observational dTRA CTO series report high access and procedural success with low severe access-site complication and radial occlusion rates.(48,49) PROGRESS-CTO data found similar technical and procedural success for dTRA and proximal radial access, acknowledging selection effects.(50)

Alternative forearm CTO strategies remain selective. The Minimalistic Hybrid registry showed comparable procedural success and a comparable composite safety endpoint with alternative forearm access versus proximal radial access, often allowing 7 Fr guide use.(51) The optimal CTO access strategy therefore depends on guide-catheter requirements, retrograde strategy, and anticipated escalation needs.(33,51)

When MCS is considered for CTO PCI, selection effects are substantial. In PROGRESS-CTO, elective MCS was used in only 4% of CTO PCIs and urgent MCS in 1%; elective MCS cases had greater clinical and angiographic complexity, similar technical and procedural success, but higher in-hospital MACE and bleeding.(86) A more recent small Impella-supported CTO/multivessel cohort similarly suggests possible feasibility, LVEF and symptom improvement, but also reported Impella-access vascular complications, underscoring that CTO-protection decisions should be individualized rather than automatic.(87)

## Severe calcification and atherectomy

Severely calcified PCI is one of the lesion subsets in which access strategy is most likely to be dictated by equipment burden. Rotational atherectomy, larger burrs, intravascular imaging, and adjunctive calcium-modification tools often require greater guide support and more generous inner-lumen capacity than routine PCI, which historically pushed many such cases toward femoral access.(34,35,71) Large-scale rotational-atherectomy data support a more nuanced approach: in a UK observational analysis of 8,622 rotational-atherectomy cases, radial access had similar 30-day mortality and procedural success but lower in-hospital major bleeding and major access-site complications than femoral access.(72)

Sheathless and rail-tracking strategies have altered this balance. In the multicentre registry by Cheaito et al., a 6.5 Fr sheathless guiding catheter supported complex PCI in small radial arteries, including kissing-balloon bifurcation PCI in 35 of 46 bifurcation lesions and rotational atherectomy in 9 patients; no radial access-site complications were reported, while two PCI failures (1.4%) were described in the detailed results.(35) Dautov et al. then extended this concept to CTO PCI with a transradial 8 Fr sheathless technique, again without excess major bleeding or vascular complications and with technical success identical to standard radial or femoral approaches (93% in each group).(34)

The most relevant contemporary calcification-specific signal comes from the SWITCH D-RIL registry. After adoption of Distal RailTracking as the default strategy for rotational atherectomy, guiding catheters sized 7 Fr or larger were used far more frequently (97.1% vs 30.7%), burrs sized 1.75 mm or larger were more common (51.4% vs 13.9%), and adjunctive plaque-modification tools were used more often (42.9% vs 24.8%), yet procedural success and short-term outcomes remained similar to the pre-DRT era.(71) These observational data suggest that access strategy may shape the lesion-preparation options available to the operator.

Not every calcified CHIP case should be forced into a forearm strategy. ERCTO data on advanced calcific plaque modification show that severe calcium within a CTO is a marker of clinical, anatomical, and procedural complexity and may require dedicated lesion-preparation devices.(17) Access should therefore be chosen for the entire lesion-preparation plan, not only for the first intended device.

## Left main and complex bifurcation PCI

Left main and complex bifurcation PCI frequently require a particularly stable platform because the procedural sequence often combines guide-catheter support, prolonged wiring, side-branch protection, kissing-balloon or two-stent techniques, and extensive intravascular imaging.(18) This does not mandate femoral access in all cases, but it lowers the threshold to favour transfemoral or enlarged-lumen forearm strategies when support, coaxiality, or device delivery are likely to be limiting.(18,32)

The issue is especially relevant when the bifurcation is embedded within CTO or heavily calcified anatomy. The JACC review on CTO and bifurcation lesions emphasizes that these subsets amplify the need for procedural planning, larger and more supportive platforms, and the ability to perform multiple sequential steps without destabilizing the guide or compromising coronary flow.(18) Accordingly, left main or complex bifurcation CHIP should be approached pragmatically: upper-extremity access is reasonable when it allows the intended guide size, imaging, and device sequence, whereas femoral access remains attractive when support needs are high or bailout options should remain straightforward.(18)

## Prior CABG and LIMA considerations

Prior CABG modifies access planning for two different reasons: the procedural anatomy is more complex, and future surgical options or conduit availability may remain clinically relevant in selected patients.(3,4,75) For CHIP, this does not mean that transradial PCI should be avoided in all post-CABG patients, but conduit preservation should enter the access discussion whenever clinically realistic.

At the same time, prior CABG often increases coronary complexity. In the LATAM CTO registry, prior CABG was associated with lower technical and procedural success and with more anatomically challenging CTO features, reinforcing that these patients often require more demanding guide support and more complex strategy planning than CABG-naïve patients.(75) Therefore, in post-CABG CHIP, access choice should balance graft anatomy, conduit preservation, support requirements, and the value of preserving alternative access routes for bailout or future procedures.(3,4,75)

## Chronic kidney disease and ultra-low-contrast workflows

In CKD and ultra-low-contrast PCI, access strategy matters mainly because it can facilitate or hinder imaging-based, contrast-sparing workflows. The access site itself is not the main determinant of renal protection, but it affects guide stability, ease of intravascular imaging, microcatheter or guide-extension use, and the ability to complete the procedure efficiently without repeated contrast confirmation.(7,76) The recent state-of-the-art review on ultra-low-contrast PCI emphasizes that ULCPCI should not be seen only as a renal-protection strategy for advanced CKD, but as a contemporary approach relevant also to multivessel disease, calcified lesions, CTO, bifurcations, severe LV dysfunction, and shock-related scenarios.(76)

That same review also reinforces several access-related practical principles that fit directly within CHIP planning: preference for patient-centred vascular access, ultrasound-guided femoral puncture when femoral access is used, marking of the common femoral bifurcation, and use of a micropuncture needle to minimise occult bleeding from repeated puncture attempts.(76) In many patients, an upper-extremity strategy aligns well with bleeding avoidance and earlier recovery, but radial access should not be pursued at the expense of procedural inefficiency if repeated catheter instability, poor support, or crossover increase overall complexity. In this scenario, the optimal access is the one that best supports a predictable imaging-guided workflow with minimal contrast need, rather than the one that is merely smaller in profile.(76)

## Obesity and peripheral artery disease

Obesity and PAD frequently push access planning in opposite directions. In obese patients, upper-extremity access may be attractive when deeper or less readily palpable femoral anatomy complicates puncture and closure.(7,23) In PAD, however, both forearm and femoral access can be compromised, making imaging-guided site selection and closure planning more important than access-site dogma.(7,13,15,16)

### Additional note on hostile femoral anatomy

In obesity and peripheral artery disease, imaging should define both puncturability and closability. Severe calcification, a small residual lumen, prior surgery, or a recent closure-device footprint may favour a modified femoral strategy, alternative access, or planned surgical collaboration rather than reflex crossover to another percutaneous route.(7,12,13,15,16,23,90)

## Cardiogenic shock and rapidly deteriorating patients

In cardiogenic shock or rapidly deteriorating patients, the idealized elective access algorithm often gives way to a simpler rule: the best access is the one that can be obtained rapidly, safely, and in a way that still permits rescue. In this context, speed and support capability may outweigh some long-term access advantages, particularly when immediate large-bore support is likely.(2,10,12) When Impella is used, single-access or alternative-access concepts described in selected elective patients may be helpful, but they should not distract from the need for a fast bailout pathway.(19,91,92) Elective VA-ECMO-supported PCI series highlight the vascular cost of aggressive support: van den Brink et al. and Shaukat et al. reported successful PCI in most or all patients, but femoral cannulation and decannulation remained central determinants of procedural risk.(96,99) The practical implication is that shock workflows should be simplified rather than idealized: if large-bore support is needed urgently, operators should prioritize the route that best combines speed, vessel suitability, and a realistic bailout plan.

Overall, the scenario-specific approach to CHIP reinforces the same principle: access strategy should be matched to the procedural mission. CTO PCI rewards flexible forearm and dual-access planning; calcified PCI rewards platforms that can support extensive lesion preparation; left main and bifurcation work require pragmatic guide stability; CKD favours efficient imaging-guided workflows; obesity and PAD demand better imaging and closure planning; and shock compresses these considerations into the shortest safe route to support and revascularisation.

## Supplementary Table S1. Practical selection of vascular access according to specific CHIP scenarios.

| **Scenario** | **Usually favoured access architecture** | **Why** | **Main caveats** |
| --- | --- | --- | --- |
| CTO PCI | Proximal radial, distal radial, dual-forearm, or femoral depending on dual-injection and guide requirements. (33,48–51,63–67,74) | Access directly shapes dual-catheter work, retrograde options, guide size, and bailout flexibility. (33) | Radial access may reduce access-site complications in selected cases, but observational radial cohorts are often less complex, and maximal support may still favour femoral access. (33,63,65–67,74) |
| Severe calcification / atherectomy | Sheathless/slender forearm when feasible; femoral when full calcium-modification burden is expected to be high. (34,35,71,72) | Guide support, larger inner lumen, intravascular imaging, and multiple devices often drive access needs. (17,18,71,72) | Do not choose access based only on the first device; plan for the whole lesion-preparation sequence. (17) |
| Left main / complex bifurcation PCI | Forearm if guide stability and imaging can be maintained; femoral when support and procedural simplicity are paramount. (18,32) | These procedures often require prolonged wiring, kissing balloons or two-stent strategies, and heavy imaging use. (18) | A radial-first strategy should not compromise coaxiality, stent delivery, or bailout options. (18,32) |
| Prior CABG / LIMA considerations | Individualised; often forearm when feasible, but with attention to conduit preservation and graft anatomy. (3,4,75) | The radial artery may retain present or future surgical value, while post-CABG PCI is often more anatomically demanding. (3,4,75) | Need to balance conduit preservation against the higher support requirements of graft or native-vessel PCI. (3,4,75) |
| CKD / ULC PCI | Access that best supports a predictable imaging-guided workflow; often forearm, but not at the cost of instability or repeated crossover. (76) | Renal protection depends on efficient IVUS/physiology-guided PCI more than on puncture site alone. (76) | A smaller access is not automatically better if it makes complex low-contrast PCI less controllable. (76) |
| Obesity / PAD | Imaging-guided individualised strategy; forearm may help in obesity, whereas hostile femoral anatomy may require planned optimisation or surgical collaboration. (7,12,13,15,16,23,90) | Deeper or less palpable femoral anatomy may complicate access in obesity; PAD affects puncture, device delivery, closure, and bailout. (7,12,13,15,16,23) | Avoid reflex crossover to another site without considering closability and rescue options. (7,12,13,15,16) |
| Cardiogenic shock / crashing patient | Fastest safe route that still permits support and rescue; often femoral if immediate large-bore MCS is likely. (2,10,12) | Speed, support capability, and bailout take priority over some long-term access advantages. (2,10,12) | The vascular price of large-bore rescue remains substantial and must be anticipated from the outset. (2,10,12) |

Note: CABG, coronary artery bypass grafting; CHIP, complex high-risk indicated percutaneous coronary intervention; CKD, chronic kidney disease; CTO, chronic total occlusion; IVUS, intravascular ultrasound; LIMA, left internal mammary artery; MCS, mechanical circulatory support; PAD, peripheral artery disease; PCI, percutaneous coronary intervention; ULC, ultra-low contrast. References are provided within table cells.

# Supplementary Appendix 2. Prevention and management of vascular complications

Access strategy should be inseparable from complication prevention. Forearm, femoral, large-bore, and MCS-related complications require mechanism-specific prevention, early recognition, and a predefined rescue pathway.(7,11,13,21–23,28,30)

## Forearm-access complications

The most relevant forearm complications in CHIP are radial artery occlusion, spasm, perforation, haematoma with rare progression to compartment syndrome, and, less commonly, nerve-related symptoms. Their incidence is strongly influenced by artery size, sheath-to-artery mismatch, anticoagulation, puncture quality, and the haemostasis technique used.(28,30,36,43,44,46,47)

Radial artery occlusion is important because it may be clinically silent yet compromise future coronary, surgical, or dialysis-related access.(28,30) DAPRAO and ANGIE reported lower forearm radial artery occlusion with dTRA but more puncture/crossover trade-offs, whereas DISCO RADIAL showed very low occlusion rates with both dTRA and conventional radial access when best practices were applied.(43,44,46)

These data imply that prevention of forearm complications begins with artery preservation principles rather than rescue maneuvers: the smallest effective outer profile, appropriate intraprocedural anticoagulation, avoidance of repeated traumatic punctures, and haemostasis strategies that preserve antegrade flow should be treated as default practice.(28,30,36,43,44,46,47) Sheathless and slender systems also contribute to prevention by reducing the sheath-to-artery ratio, which in the randomised comparison by Horie et al. was independently associated with the composite of radial artery occlusion and severe radial artery spasm.(36)

Management should distinguish spasm from haematoma. Radial spasm is treated with analgesia and sedation, intra-arterial vasodilators, forearm warming, and reactive hyperaemia.(30) A limited, non-expanding forearm haematoma requires prompt compression at the bleeding source, elevation of the arm above heart level, and serial neurovascular assessment.(30,77) Antithrombotic interruption or adjustment should not be automatic; it should be individualised for clinically significant or expanding bleeding after balancing bleeding severity and procedural circumstances against thrombotic risk.(77) Pharmacological reversal should likewise not be routine and should be individualised to the bleeding mechanism, severity, anticoagulant, and thrombotic risk.(78) Progressive swelling, severe pain, paraesthesia, motor deficit, or tense compartments should trigger urgent evaluation for perforation or compartment syndrome.(30,77)

## Femoral and large-bore complications

Femoral complications in CHIP range from venipuncture and haematoma to pseudoaneurysm, arteriovenous fistula, retroperitoneal bleeding, dissection, closure-device failure, infection, and limb ischaemia in large-bore settings. Suboptimal puncture location is best addressed by image-guided common femoral access,(7,23) whereas large-bore sheath use, hostile anatomy, and closure-device failure require a preplanned closure and bailout strategy.(12,13,15,26)

The preventive value of image-guided puncture is best established by FAUST. Compared with fluoroscopic guidance alone, ultrasound guidance improved first-pass success, reduced the number of attempts, reduced accidental venipuncture, and lowered vascular complications from 3.4% to 1.4%.(23) These findings are particularly relevant in CHIP because the consequences of even a modestly misplaced puncture become magnified once larger sheath sizes, anticoagulation, prolonged procedures, or closure devices are added.(7,11,23)

In large-bore access, closure-device failure becomes central. MASH showed similar vascular complications and clinically relevant bleeding with MANTA and double ProGlide, but less modified closure-device failure with MANTA and different bailout patterns.(11) Barbanti et al. likewise showed that vascular complications and closure failure vary by device strategy.(89) These data emphasize that large-bore risk depends on closure and rescue, not puncture alone.

The most practical bailout data come from Eitan et al. In their single-centre series, 11 patients undergoing transfemoral TAVR in whom pre-closure could not be performed underwent planned covered-stent implantation for access-site haemostasis. Haemostasis was achieved in all cases, 30-day mortality was 0%, and no patient required additional vascular interventions during follow-up.(26) This paper is valuable not because covered stents should become routine, but because it demonstrates that percutaneous bailout can be effective when it is anticipated and technically supported.

Hostile femoral anatomy should trigger preventive planning rather than late rescue. Heavy calcification, a small residual lumen, and an unfavourable closure zone may justify alternative access, a modified femoral strategy, or planned surgical collaboration. Evidence for vessel-preparation techniques remains limited and should be interpreted cautiously.(7,12,13,15,16,90)

## MCS-related vascular complications

MCS-related vascular complications should be treated as predictable access penalties rather than unexpected rare events. Impella-supported PCI is associated with large-bore arterial injury and bleeding risk.(14) VA-ECMO adds venous cannulation, decannulation, and limb-perfusion concerns.(96–99) iVAC 2L remains a large-bore femoral strategy despite favourable contemporary registry signals.(102–104)

For Impella-supported PCI, Johannsen et al. provide one of the clearest practical estimates. Among 61 high-risk PCI patients treated with Impella using a standardised protocol of angiographic- and fluoroscopic-guided femoral access, major vascular complications occurred in 5 patients (8%). All were managed conservatively, and peripheral arterial disease was associated with major vascular complications in regression analysis.(14) These findings support careful patient selection and image-guided femoral access, while showing that vascular risk is not eliminated by a standardised protocol.

VA-ECMO carries a broader access burden because it adds venous cannulation, decannulation complexity, and limb-perfusion concerns. Across elective series, surgical arterial repair and haematoma were reported by Shaukat et al.(96); limb ischaemia, haematoma, transfusion, and dialysis were reported by Ungureanu et al.(97); and femoral-vein thrombosis was reported by van den Brink et al.(99)

For iVAC 2L, the contemporary registry signal appears somewhat more favourable, but the access burden remains real. In the international iVAC 2L Registry of 293 high-risk PCI cases, major vascular complications occurred in 2.1% and major bleeding in 1.0%, with no urgent cardiac or vascular surgery required for the major vascular events reported. However, one patient with major bleeding died before the intended surgical intervention could be performed.(104) These outcomes are reassuring, but they still locate iVAC 2L within the domain of large-bore femoral planning rather than as a low-access-burden support option.

The practical implication across all support platforms is that MCS-related complication prevention begins before insertion: vessel assessment, ultrasound-guided puncture, appropriate anticoagulation, predefined closure strategy, preservation of guidewire access until haemostasis is secure, selective preparation of calcified iliofemoral segments when justified, and early consideration of distal perfusion or crossover control when limb flow is at risk.(12,13,15,90) Once the complication occurs, successful rescue usually depends on whether these elements were already anticipated.

## Practical rescue and escalation pathways

For forearm complications, treat spasm with analgesia and sedation, intra-arterial vasodilators, forearm warming, and reactive hyperaemia.(30) Treat a limited, non-expanding haematoma with direct compression at the bleeding source, elevation of the arm above heart level, and serial neurovascular assessment.(30,77) Antithrombotic interruption or adjustment should not be automatic; it should be individualised for clinically significant or expanding bleeding after balancing bleeding severity and procedural circumstances against thrombotic risk.(77) Pharmacological reversal should likewise not be routine and should be individualised to the bleeding mechanism, severity, anticoagulant, and thrombotic risk.(78) Expanding haematoma, persistent pain, or neurological symptoms should prompt urgent reassessment for perforation or compartment syndrome.(28,30,77)

For femoral complications, early angiographic clarification is often decisive. Balloon tamponade, crossover control, covered stent implantation, or surgical conversion should be selected according to the mechanism of failure and the anatomical substrate.(15,26)

In large-bore or MCS-related complications, prolonged indecision is often more dangerous than early escalation. A limited dissection, persistent bleeding after closure failure, or threatened limb perfusion may still be managed endovascularly if a secondary access route and wire control are maintained; once these are lost, surgical rescue becomes more likely.(15,26) This is why pre-closure planning, retention of access until haemostasis is confirmed, and low threshold for post-procedural imaging are not merely technical refinements but part of a complication-management philosophy.

Overall, complication management in CHIP should be framed as a continuum: anticipate, prevent, recognise early, and escalate decisively. The success of a complex PCI is not complete until the vascular access has also been safely concluded.

## Supplementary Table S2. Prevention and management of forearm, femoral, large-bore, and MCS-related vascular complications.

| **Complication domain** | **Key preventive measures** | **Early warning signs** | **Initial bailout / escalation** |
| --- | --- | --- | --- |
| Forearm access (radial / distal radial / ulnar) | Smallest effective outer profile; adequate anticoagulation; minimising traumatic puncture; patent haemostasis; artery-preservation mindset. (24,25,28,30,36,43–47) | Progressive swelling, severe pain, loss of pulse, persistent spasm, neurological symptoms, suspected perforation or compartment syndrome. (28,30,77) | Spasm: analgesia and sedation, intra-arterial vasodilators, forearm warming, and reactive hyperaemia. Haematoma: direct compression at the bleeding source, elevation of the arm above heart level, and serial neurovascular assessment. Antithrombotic interruption or adjustment should not be automatic; individualise it for clinically significant or expanding bleeding after balancing bleeding severity and procedural circumstances against thrombotic risk. Pharmacological reversal should likewise not be routine and should be individualised to the bleeding mechanism, severity, anticoagulant, and thrombotic risk. Urgently evaluate for perforation or compartment syndrome if symptoms progress. (7,28,30,77,78) |
| Standard femoral access | Ultrasound-guided common femoral puncture; anterior-wall puncture; micropuncture; early angiographic confirmation when needed. (7,12,15,21–23,73) | Groin pain or swelling, persistent bleeding, falling haemoglobin, suspicion of pseudoaneurysm, AV fistula, or retroperitoneal bleed. (7,12,15) | Immediate clarification of the puncture problem; compression, angiographic assessment, and escalation according to mechanism. (12,15) |
| Large-bore femoral access / closure failure | Preprocedural vessel assessment; planned pre-closure when appropriate; maintain wire control until haemostasis is secure; know the bailout pathway before insertion. (11,12,15,89) | Persistent bleeding after closure, loss of haemostasis, flow-limiting dissection, threatened limb perfusion, device-deployment failure. (11,12,15,89) | Balloon tamponade, crossover control, additional closure device if appropriate, covered stent, or surgical conversion according to anatomy and mechanism. (11,15,26) |
| Hostile femoral anatomy | Assess not only puncturability but closability; identify heavy calcification, small lumen, or unfavourable closure zone before large-bore steps. (13,15,16,90) | Closure-device footplate or needle failure, repeated inability to deploy suture-mediated closure, impaired vessel compliance. (13,15,16,90) | Consider a modified femoral strategy or selective vessel preparation only in expert centres, recognising the limited evidence; otherwise use alternative access or early surgical collaboration. (13,15,16,90) |
| MCS-related complications | Plan puncture, closure, surveillance, and bailout before support insertion; consider limb-perfusion protection when relevant. (2,10,12,14,100,101) | Bleeding, transfusion requirement, limb ischaemia, haemodynamic instability after device removal, venous complications in ECMO. (2,10,12,14,99–101) | Low threshold for angiography or duplex imaging, maintenance of access until haemostasis is confirmed, and early endovascular or surgical escalation when flow or haemostasis is threatened. (2,10,12,14,100,101) |

Note: AV, arteriovenous; ECMO, extracorporeal membrane oxygenation; MCS, mechanical circulatory support. References are provided within table cells.

# REFERENCES CITED IN THE SUPPLEMENTARY MATERIAL

Numbering is retained from the revised main manuscript.

2. Ferro EG, Kim JM, Lalani C, Abbott DJ, Yeh RW. Mechanical circulatory support for complex, high-risk percutaneous coronary intervention. EuroIntervention. 2025 Feb 3;21(3):e149-e160. doi: 10.4244/EIJ-D-24-00386. PMID: 39901631; PMCID: PMC11776407.

3. Neumann FJ, Sousa-Uva M, Ahlsson A, Alfonso F, Banning AP, Benedetto U, et al. 2018 ESC/EACTS Guidelines on myocardial revascularization. Eur Heart J. 2019 Jan 7;40(2):87-165. doi: 10.1093/eurheartj/ehy394. Erratum in: Eur Heart J. 2019 Oct 1;40(37):3096. doi: 10.1093/eurheartj/ehz507. PMID: 30165437.

4. Lawton JS, Tamis-Holland JE, Bangalore S, Bates ER, Beckie TM, Bischoff JM, et al. 2021 ACC/AHA/SCAI Guideline for Coronary Artery Revascularization: A Report of the American College of Cardiology/American Heart Association Joint Committee on Clinical Practice Guidelines. Circulation. 2022 Jan 18;145(3):e18-e114. doi: 10.1161/CIR.0000000000001038. Epub 2021 Dec 9. Erratum in: Circulation. 2022 Mar 15;145(11):e772. doi: 10.1161/CIR.0000000000001060. PMID: 34882435.

7. Sandoval Y, Burke MN, Lobo AS, Lips DL, Seto AH, Chavez I, et al. Contemporary Arterial Access in the Cardiac Catheterization Laboratory. JACC Cardiovasc Interv. 2017 Nov 27;10(22):2233-2241. doi: 10.1016/j.jcin.2017.08.058. PMID: 29169493.

10. Chieffo A, Dudek D, Hassager C, Combes A, Gramegna M, Halvorsen S, et al. Joint EAPCI/ACVC expert consensus document on percutaneous ventricular assist devices. EuroIntervention. 2021 Jul 20;17(4):e274-e286. doi: 10.4244/EIJY21M05_01. PMID: 34057071; PMCID: PMC9709772.

11. van Wiechen MP, Tchétché D, Ooms JF, Hokken TW, Kroon H, Ziviello F, et al. Suture- or Plug-Based Large-Bore Arteriotomy Closure: A Pilot Randomized Controlled Trial. JACC Cardiovasc Interv. 2021 Jan 25;14(2):149-157. doi: 10.1016/j.jcin.2020.09.052. Epub 2020 Dec 23. PMID: 33358648.

12. Sandoval Y, Basir MB, Lemor A, Lichaa H, Alasnag M, Dupont A, et al. Optimal Large-Bore Femoral Access, Indwelling Device Management, and Vascular Closure for Percutaneous Mechanical Circulatory Support. Am J Cardiol. 2023 Nov 1;206:262-276. doi: 10.1016/j.amjcard.2023.08.024. Epub 2023 Sep 15. PMID: 37717476.

13. Spano' A, De Backer O, Amat-Santos IJ, Boukhris M, Jossart A, Auslender J, et al. Angioplasty-Assisted, Suture-Mediated Vascular Closure Enabling Percutaneous Transfemoral TAVR in Case of a Diseased Puncture Site. The FEM-PREP Technique. Catheter Cardiovasc Interv. 2026 Jan;107(1):61-69. doi: 10.1002/ccd.70285. Epub 2025 Nov 2. PMID: 41178299.

14. Johannsen L, Mahabadi AA, Totzeck M, Krueger A, Jánosi RA, Rassaf T, et al. Access site complications following Impella-supported high-risk percutaneous coronary interventions. Sci Rep. 2019 Nov 28;9(1):17844. doi: 10.1038/s41598-019-54277-w. PMID: 31780769; PMCID: PMC6882834.

15. Paraggio L, Bianchini F, Aurigemma C, Romagnoli E, Bianchini E, Zito A, et al. Femoral Large Bore Sheath Management: How to Prevent Vascular Complications From Vessel Puncture to Sheath Removal. Circ Cardiovasc Interv. 2024 Sep;17(9):e014156. doi: 10.1161/CIRCINTERVENTIONS.124.014156. Epub 2024 Aug 21. PMID: 39166330; PMCID: PMC11404769.

16. Cheney AE, McCabe JM. Alternative Percutaneous Access for Large Bore Devices. Circ Cardiovasc Interv. 2019 Jun;12(6):e007707. doi: 10.1161/CIRCINTERVENTIONS.118.007707. Epub 2019 Jun 6. PMID: 31167600.

17. Vadalà G, Mashayekhi K, Behnes M, Ayoub M, Gorgulu S, Werner GS, et al. EURO CTO Investigators. Procedural Impact of Advanced Calcific Plaque Modification Devices Within Percutaneous Revascularization of Chronic Total Occlusions. JACC Cardiovasc Interv. 2025 Jun 9;18(11):1376-1390. doi: 10.1016/j.jcin.2025.04.035. PMID: 40500006.

18. Lefèvre T, Pan M, Stankovic G, Ojeda S, Boudou N, Brilakis ES, et al. CTO and Bifurcation Lesions: An Expert Consensus From the European Bifurcation Club and EuroCTO Club. JACC Cardiovasc Interv. 2023 Sep 11;16(17):2065-2082. doi: 10.1016/j.jcin.2023.06.042. PMID: 37704294.

19. Wollmuth J, Korngold E, Croce K, Pinto DS. The Single-access for Hi-risk PCI (SHiP) technique. Catheter Cardiovasc Interv. 2020 Jul;96(1):114-116. doi: 10.1002/ccd.28556. Epub 2019 Oct 26. PMID: 31654483; PMCID: PMC7383894.

21. d'Entremont MA, Alrashidi S, Seto AH, Nguyen P, Marquis-Gravel G, Abu-Fadel MS, et al. Ultrasound guidance for transfemoral access in coronary procedures: an individual participant-level data metaanalysis from the femoral ultrasound trialist collaboration. EuroIntervention. 2024 Jan 1;20(1):66-74. doi: 10.4244/EIJ-D-22-00809. PMID: 37800723; PMCID: PMC10758987.

22. d'Entremont MA, Seto AH, Alrashidi S, Alansari O, Brochu B, Lemaire-Paquette S, et al. Ultrasound-Guided Transfemoral Access for Coronary Procedures: A Pooled Learning Curve Analysis From the FAUST and UNIVERSAL Trials. Circ Cardiovasc Interv. 2024 Aug;17(8):e013817. doi: 10.1161/CIRCINTERVENTIONS.123.013817. Epub 2024 Jun 18. PMID: 38887948.

23. Seto AH, Abu-Fadel MS, Sparling JM, Zacharias SJ, Daly TS, Harrison AT, et al. Real-time ultrasound guidance facilitates femoral arterial access and reduces vascular complications: FAUST (Femoral Arterial Access With Ultrasound Trial). JACC Cardiovasc Interv. 2010 Jul;3(7):751-8. doi: 10.1016/j.jcin.2010.04.015. PMID: 20650437.

24. Seto AH, Roberts JS, Abu-Fadel MS, Czak SJ, Latif F, Jain SP, et al. Real-time ultrasound guidance facilitates transradial access: RAUST (Radial Artery access with Ultrasound Trial). JACC Cardiovasc Interv. 2015 Feb;8(2):283-291. doi: 10.1016/j.jcin.2014.05.036. Epub 2015 Jan 14. PMID: 25596790.

25. Bianchini F, Lombardi M, Ricchiuto A, Paraggio L, Aurigemma C, Romagnoli E, et al. Combined ultrasound and angiographic guidance to facilitate transradial access procedures. Catheter Cardiovasc Interv. 2024 Feb;103(3):443-454. doi: 10.1002/ccd.30947. Epub 2024 Jan 3. PMID: 38173287.

26. Eitan A, Shiran A, Sliman H, Zafrir B, Jaffe R. Totally Percutaneous Transfemoral Transcatheter Aortic Valve Replacement Despite Failure to Deploy a Vascular Closure Device: A Single-Centre Case Series. Heart Lung Circ. 2022 Mar;31(3):390-394. doi: 10.1016/j.hlc.2021.09.005. Epub 2021 Oct 1. PMID: 34607752.

28. Shroff AR, Gulati R, Drachman DE, Feldman DN, Gilchrist IC, Kaul P, et al. SCAI expert consensus statement update on best practices for transradial angiography and intervention. Catheter Cardiovasc Interv. 2020 Feb;95(2):245-252. doi: 10.1002/ccd.28672. Epub 2019 Dec 27. PMID: 31880380.

29. Rao SV, Cohen MG, Kandzari DE, Bertrand OF, Gilchrist IC. The transradial approach to percutaneous coronary intervention: historical perspective, current concepts, and future directions. J Am Coll Cardiol. 2010 May 18;55(20):2187-95. doi: 10.1016/j.jacc.2010.01.039. PMID: 20466199.

30. Mason PJ, Shah B, Tamis-Holland JE, Bittl JA, Cohen MG, Safirstein J, et al. American Heart Association Interventional Cardiovascular Care Committee of the Council on Clinical Cardiology; Council on Cardiovascular and Stroke Nursing; Council on Peripheral Vascular Disease; and Council on Genomic and Precision Medicine. An Update on Radial Artery Access and Best Practices for Transradial Coronary Angiography and Intervention in Acute Coronary Syndrome: A Scientific Statement From the American Heart Association. Circ Cardiovasc Interv. 2018 Sep;11(9):e000035. doi: 10.1161/HCV.0000000000000035. PMID: 30354598.

31. Gargiulo G, Giacoppo D, Jolly SS, Cairns J, Le May M, Bernat I, et al. Effects on Mortality and Major Bleeding of Radial Versus Femoral Artery Access for Coronary Angiography or Percutaneous Coronary Intervention: Meta-Analysis of Individual Patient Data From 7 Multicenter Randomized Clinical Trials. Circulation. 2022 Nov;146(18):1329-1343. doi: 10.1161/CIRCULATIONAHA.122.061527. Epub 2022 Aug 29. PMID: 36036610.

32. Meijers TA, Aminian A, van Wely M, Teeuwen K, Schmitz T, Dirksen MT, et al. Randomized Comparison Between Radial and Femoral Large-Bore Access for Complex Percutaneous Coronary Intervention. JACC Cardiovasc Interv. 2021 Jun 28;14(12):1293-1303. doi: 10.1016/j.jcin.2021.03.041. Epub 2021 May 18. PMID: 34020929.

33. Meijers TA, Aminian A, Valgimigli M, Dens J, Agostoni P, Iglesias JF, et al. Vascular Access in Percutaneous Coronary Intervention of Chronic Total Occlusions: A State-of-the-Art Review. Circ Cardiovasc Interv. 2023 Aug;16(8):e013009. doi: 10.1161/CIRCINTERVENTIONS.123.013009. Epub 2023 Jul 17. PMID: 37458110.

34. Dautov R, Ribeiro HB, Abdul-Jawad Altisent O, Nombela-Franco L, Gibrat C, et al. Effectiveness and Safety of the Transradial 8Fr Sheathless Approach for Revascularization of Chronic Total Occlusions. Am J Cardiol. 2016 Sep 15;118(6):785-789. doi: 10.1016/j.amjcard.2016.06.052. Epub 2016 Jun 28. PMID: 27453512.

35. Cheaito R, Benamer H, Hovasse T, Tritar A, Hage F, Garot P, et al. Feasibility and safety of transradial coronary interventions using a 6.5-F sheathless guiding catheter in patients with small radial arteries. Catheter Cardiovasc Interv. 2015 Jul;86(1):51-8. doi: 10.1002/ccd.25508. Epub 2015 Mar 30. PMID: 24740683.

36. Horie K, Tada N, Isawa T, Matsumoto T, Taguri M, Kato S, et al. A randomised comparison of incidence of radial artery occlusion and symptomatic radial artery spasm associated with elective transradial coronary intervention using 6.5 Fr SheathLess Eaucath Guiding Catheter vs. 6.0 Fr Glidesheath Slender. EuroIntervention. 2018 Apr 20;13(17):2018-2025. doi: 10.4244/EIJ-D-17-00239. PMID: 28846541.

42. Sgueglia GA, Di Giorgio A, Gaspardone A, Babunashvili A. Anatomic Basis and Physiological Rationale of Distal Radial Artery Access for Percutaneous Coronary and Endovascular Procedures. JACC Cardiovasc Interv. 2018 Oct 22;11(20):2113-2119. doi: 10.1016/j.jcin.2018.04.045. PMID: 30336816.

43. Eid-Lidt G, Rivera Rodríguez A, Jimenez Castellanos J, Farjat Pasos JI, Estrada López KE, Gaspar J. Distal Radial Artery Approach to Prevent Radial Artery Occlusion Trial. JACC Cardiovasc Interv. 2021 Feb 22;14(4):378-385. doi: 10.1016/j.jcin.2020.10.013. PMID: 33602433.

44. Tsigkas G, Papageorgiou A, Moulias A, Kalogeropoulos AP, Papageorgopoulou C, Apostolos A, et al. Distal or Traditional Transradial Access Site for Coronary Procedures: A Single-Center, Randomized Study. JACC Cardiovasc Interv. 2022 Jan 10;15(1):22-32. doi: 10.1016/j.jcin.2021.09.037. Epub 2021 Dec 15. PMID: 34922888.

45. Ferrante G, Condello F, Rao SV, Maurina M, Jolly S, Stefanini GG, et al. Distal vs Conventional Radial Access for Coronary Angiography and/or Intervention: A Meta-Analysis of Randomized Trials. JACC Cardiovasc Interv. 2022 Nov 28;15(22):2297-2311. doi: 10.1016/j.jcin.2022.09.006. PMID: 36423974.

46. Aminian A, Sgueglia GA, Wiemer M, Kefer J, Gasparini GL, Ruzsa Z, et al. Distal Versus Conventional Radial Access for Coronary Angiography and Intervention: The DISCO RADIAL Trial. JACC Cardiovasc Interv. 2022 Jun 27;15(12):1191-1201. doi: 10.1016/j.jcin.2022.04.032. Epub 2022 May 17. PMID: 35595673.

47. Schenke K, Prog R, Joghetaei N, Sood N, Viertel A, Matthiesen T, et al. CORRECT RADIAL RCT. CORonaRy angiography and intErventions via distal vs proximal transradial aCcess-a randomized Trial of different RADIAL puncture sites. Clin Res Cardiol. 2026 Jan;115(1):99-111. doi: 10.1007/s00392-025-02759-5. Epub 2025 Nov 24. PMID: 41284047; PMCID: PMC12783259.

48. Achim A, Szigethy T, Olajos D, Molnár L, Papp R, Bárczi G, et al. Switching From Proximal to Distal Radial Artery Access for Coronary Chronic Total Occlusion Recanalization. Front Cardiovasc Med. 2022 May 9;9:895457. doi: 10.3389/fcvm.2022.895457. PMID: 35615565; PMCID: PMC9124806.

49. Lin CJ, Lee WC, Lee CH, Chung WJ, Hsueh SK, Chen CJ, Yang CH, Fang HY, Cheng CI, Wu CJ. Feasibility and Safety of Chronic Total Occlusion Percutaneous Coronary Intervention via Distal Transradial Access. Front Cardiovasc Med. 2021 May 10;8:673858. doi: 10.3389/fcvm.2021.673858. PMID: 34041286; PMCID: PMC8141614.

50. Nikolakopoulos I, Patel T, Jefferson BK, Sheikh AM, Jaber W, Samady H, et al. Distal Radial Access in Chronic Total Occlusion Percutaneous Coronary Intervention: Insights From the PROGRESS-CTO Registry. J Invasive Cardiol. 2021 Sep;33(9):E717-E722. doi: 10.25270/jic/20.00300. Epub 2021 Aug 19. PMID: 34433693.

51. Poletti E, Castaldi G, Scott B, Benedetti A, Moroni A, El Jattari H, et al. Alternative (Transulnar or Distal Radial) Arterial Access for Chronic Total Occlusion Percutaneous Coronary Intervention (Subanalysis from the Minimalistic Hybrid Approach Algorithm Registry). Am J Cardiol. 2023 Aug 1;200:57-65. doi: 10.1016/j.amjcard.2023.05.005. Epub 2023 Jun 9. PMID: 37300902.

56. Gokhroo RK, Chandra K, Nandal R, Bisht DS, Gupta S, Kishor K, et al. The initial experience of 2495 cases of the ulnar artery as default access for coronary diagnostic and interventional procedures at a single center: An observational study. Indian Heart J. 2020 May-Jun;72(3):184-188. doi: 10.1016/j.ihj.2020.05.010. Epub 2020 Jun 14. PMID: 32768018; PMCID: PMC7411112.

57. Hahalis G, Tsigkas G, Xanthopoulou I, Deftereos S, Ziakas A, Raisakis K, et al. Transulnar compared with transradial artery approach as a default strategy for coronary procedures: a randomized trial. The Transulnar or Transradial Instead of Coronary Transfemoral Angiographies Study (the AURA of ARTEMIS Study). Circ Cardiovasc Interv. 2013 Jun;6(3):252-61. doi: 10.1161/CIRCINTERVENTIONS.112.000150. Epub 2013 Jun 4. PMID: 23735472.

58. Gokhroo R, Kishor K, Ranwa B, Bisht D, Gupta S, Padmanabhan D, et al. Ulnar Artery Interventions Non-Inferior to Radial Approach: AJmer Ulnar ARtery (AJULAR) Intervention Working Group Study Results. J Invasive Cardiol. 2016 Jan;28(1):1-8. PMID: 26716587.

59. Karelas D, Aznaouridis K, Manousopoulos K, Filippou K, Varelas P, Papadopoulos I, et al. Transulnar access in patients with ipsilateral radial artery occlusion undergoing coronary angiography or angioplasty: Rationale and design of the ULNART study. Cardiovasc Revasc Med. 2026 Apr 3:S1553-8389(26)00135-1. doi: 10.1016/j.carrev.2026.04.001. Epub ahead of print. PMID: 41968006.

60. Ozcinar E, Dikmen N, Kayan A, Baran C, Yazicioglu L. Adverse Events in Open Surgical vs. Ultrasound-Guided Percutaneous Brachial Access for Endovascular Interventions. J Clin Med. 2024 Jul 17;13(14):4179. doi: 10.3390/jcm13144179. PMID: 39064219; PMCID: PMC11277864.

61. Destrieux L; A.R.C.H.I.V. Group. Safety and Effectiveness of Ultrasound-Guided Percutaneous Versus Open Brachial Artery Access: Results of the Multicenter Prospective ARCHIBAL Study Percutaneous or Open Brachial Artery Access. Ann Vasc Surg. 2025 Feb;111:231-240. doi: 10.1016/j.avsg.2024.10.015. Epub 2024 Nov 23. PMID: 39586528.

62. Protty M, Gallagher S, Sharp ASP, Anderson R, Ludman P, Curzen N, et al. Brachial arterial access for PCI: an analysis of the British Cardiovascular Intervention Society database. EuroIntervention. 2022 Jan 28;17(13):1100-1103. doi: 10.4244/EIJ-D-21-00294. PMID: 34278994; PMCID: PMC9724922.

63. Kinnaird T, Anderson R, Ossei-Gerning N, Gallagher S, Large A, Strange J, et al. Vascular Access Site and Outcomes Among 26,807 Chronic Total Coronary Occlusion Angioplasty Cases From the British Cardiovascular Interventions Society National Database. JACC Cardiovasc Interv. 2017 Apr 10;10(7):635-644. doi: 10.1016/j.jcin.2016.11.055. PMID: 28385401.

64. Tajti P, Alaswad K, Karmpaliotis D, Jaffer FA, Yeh RW, Patel M, et al. Procedural Outcomes of Percutaneous Coronary Interventions for Chronic Total Occlusions Via the Radial Approach: Insights From an International Chronic Total Occlusion Registry. JACC Cardiovasc Interv. 2019 Feb 25;12(4):346-358. doi: 10.1016/j.jcin.2018.11.019. PMID: 30784639.

65. Megaly M, Karatasakis A, Abraham B, Jensen J, Saad M, Omer M, et al. Radial Versus Femoral Access in Chronic Total Occlusion Percutaneous Coronary Intervention. Circ Cardiovasc Interv. 2019 Jun;12(6):e007778. doi: 10.1161/CIRCINTERVENTIONS.118.007778. Epub 2019 Jun 14. PMID: 31195826.

66. Chakravartti J, Feser WJ, Plomondon ME, Valle JA, Rao SV, Gutierrez JA, et al. Access Site Selection and Outcomes for Chronic Total Occlusion Percutaneous Coronary Interventions: Insights from the VA CART Program. J Soc Cardiovasc Angiogr Interv. 2022 Aug 24;1(6):100440. doi: 10.1016/j.jscai.2022.100440. PMID: 39132361; PMCID: PMC11308032.

67. Gorgulu S, Kalay N, Norgaz T, Kocas C, Goktekin O, Brilakis ES. Femoral or Radial Approach in Treatment of Coronary Chronic Total Occlusion: A Randomized Clinical Trial. JACC Cardiovasc Interv. 2022 Apr 25;15(8):823-830. doi: 10.1016/j.jcin.2022.02.012. Erratum in: JACC Cardiovasc Interv. 2022 Jul 11;15(13):1396. doi: 10.1016/j.jcin.2022.06.002. PMID: 35450683.

71. Colletti G, Sgueglia GA, Jossart A, Lepièce C, Gach O, Natalis A, et al. SWITCHing to Distal RaiLtracking technique for rotational atherectomy procedures: short-term outcomes and impact on clinical practice (the SWITCH D-RIL registry). J Invasive Cardiol. 2026 Jan 26. doi: 10.25270/jic/25.00355. Epub ahead of print. PMID: 41592160.

72. Watt J, Austin D, Mackay D, Nolan J, Oldroyd KG. Radial Versus Femoral Access for Rotational Atherectomy: A UK Observational Study of 8622 Patients. Circ Cardiovasc Interv. 2017 Dec;10(12):e005311. doi: 10.1161/CIRCINTERVENTIONS.117.005311. PMID: 29246910.

73. Meijers TA, Nap A, Aminian A, Schmitz T, Dens J, Teeuwen K, et al. Ultrasound-guided versus fluoroscopy-guided large-bore femoral access in PCI of complex coronary lesions: the international, multicentre, randomised ULTRACOLOR Trial. EuroIntervention. 2024 Jul 15;20(14):e876-e886. doi: 10.4244/EIJ-D-24-00089. PMID: 38742577; PMCID: PMC11228538.

74. Simsek B, Gorgulu S, Kostantinis S, Karacsonyi J, Alaswad K, Jaffer FA, et al. Radial access for chronic total occlusion percutaneous coronary intervention: Insights from the PROGRESS-CTO registry. Catheter Cardiovasc Interv. 2022 Nov;100(5):730-736. doi: 10.1002/ccd.30347. Epub 2022 Jul 23. PMID: 35870177.

75. Hernandez-Suarez DF, Azzalini L, Moroni F, Tinoco de Paula JE, Lamelas P, Campos CM, et al. Outcomes of chronic total occlusion percutaneous coronary intervention in patients with prior coronary artery bypass graft surgery: Insights from the LATAM CTO registry. Catheter Cardiovasc Interv. 2022 Feb;99(2):245-253. doi: 10.1002/ccd.30041. Epub 2021 Dec 20. PMID: 34931448; PMCID: PMC8885848.

76. Shabbir A, Ali Z, Colletti G, Dudek D, Garbo R, Hellig F, et al. Ultra-Low-Contrast PCI: A Structured Approach to Reducing Dependence on Contrast Vessel Opacification in PCI. JACC Cardiovasc Interv. 2025 Feb 24;18(4):409-424. doi: 10.1016/j.jcin.2024.11.043. PMID: 40010912.

77. Zhi G, Lei M, Qian S, Zhang C, Li Y, Zhao Z, et al. Nursing of forearm hematoma after transradial coronary intervention. Health Sci Rep. 2024;7(9):e70050. doi: 10.1002/hsr2.70050. PMID: 39221046; PMCID: PMC11362833.

78. Sawant AC, Rizik DG, Rao SV, Pershad A. Algorithms for challenging scenarios encountered in transradial intervention. Indian Heart J. 2021;73(2):149-155. doi: 10.1016/j.ihj.2020.09.012. PMID: 33865510; PMCID: PMC8065373.

86. Danek BA, Basir MB, O'Neill WW, Alqarqaz M, Karatasakis A, Karmpaliotis D, et al. Mechanical Circulatory Support in Chronic Total Occlusion Percutaneous Coronary Intervention: Insights From a Multicenter U.S. Registry. J Invasive Cardiol. 2018 Mar;30(3):81-87. PMID: 29493509.

87. Gallo I, Azzalini L, González-Manzanares R, Moscardelli S, Jurado-Román A, Maestre LC, et al. Mechanical Circulatory Support With Impella in High-Risk Patients With Chronic Total Occlusion and Complex Multivessel Disease. Catheter Cardiovasc Interv. 2025 Mar;105(4):883-890. doi: 10.1002/ccd.31392. Epub 2025 Jan 8. PMID: 39777977; PMCID: PMC11874245.

89. Barbanti M, Capranzano P, Ohno Y, Gulino S, Sgroi C, Immè S, et al. Comparison of suture-based vascular closure devices in transfemoral transcatheter aortic valve implantation. EuroIntervention. 2015 Oct;11(6):690-7. doi: 10.4244/EIJV11I6A137. PMID: 26499222.

90. Riley RF, Corl JD, Kereiakes DJ. Intravascular lithotripsy-assisted Impella insertion: A case report. Catheter Cardiovasc Interv. 2019 Jun 1;93(7):1317-1319. doi: 10.1002/ccd.28168. Epub 2019 Feb 27. PMID: 30811781.

91. Truong HTD, Hunter G, Lotun K, Shetty R, Shanmugasundaram M, Kapoor D, et al. Insertion of the Impella via the axillary artery for high-risk percutaneous coronary intervention. Cardiovasc Revasc Med. 2018 Jul;19(5 Pt A):540-544. doi: 10.1016/j.carrev.2017.12.016. Epub 2018 Jan 5. PMID: 29422276.

92. McCabe JM, Kaki AA, Pinto DS, Kirtane AJ, Nicholson WJ, Grantham JA, et al. Percutaneous Axillary Access for Placement of Microaxial Ventricular Support Devices: The Axillary Access Registry to Monitor Safety (ARMS). Circ Cardiovasc Interv. 2021 Jan;14(1):e009657. doi: 10.1161/CIRCINTERVENTIONS.120.009657. Epub 2020 Dec 16. PMID: 33322918; PMCID: PMC7813449.

96. Shaukat A, Hryniewicz-Czeneszew K, Sun B, Mudy K, Wilson K, Tajti P, et al. Outcomes of Extracorporeal Membrane Oxygenation Support for Complex High-Risk Elective Percutaneous Coronary Interventions: A Single-Center Experience and Review of the Literature. J Invasive Cardiol. 2018 Dec;30(12):456-460. PMID: 30504514.

97. Ungureanu C, Blaimont M, Trine H, Henin P, Courcelle R, Laurent Y, et al. Prophylactic ECMO Support during Elective Coronary Percutaneous Interventions in High-Risk Patients: A Single-Center Experience. J Interv Cardiol. 2023 Feb 4;2023:5332038. doi: 10.1155/2023/5332038. PMID: 36793669; PMCID: PMC9922177.

98. Tomasello SD, Boukhris M, Ganyukov V, Galassi AR, Shukevich D, Haes B, et al. Outcome of extracorporeal membrane oxygenation support for complex high-risk elective percutaneous coronary interventions: A single-center experience. Heart Lung. 2015 Jul-Aug;44(4):309-13. doi: 10.1016/j.hrtlng.2015.03.005. Epub 2015 Apr 23. PMID: 25913808.

99. Van den Brink FS, Meijers TA, Hofma SH, van Boven AJ, Nap A, Vonk A, et al. Prophylactic veno-arterial extracorporeal membrane oxygenation in patients undergoing high-risk percutaneous coronary intervention. Neth Heart J. 2020 Mar;28(3):139-144. doi: 10.1007/s12471-019-01350-8. PMID: 31782108; PMCID: PMC7052097.

100. Griffioen AM, van Wely MH, van Nunen LX, van den Oord SCH, van Wetten HB, de Jong SF, et al. Outcomes of high-risk PCI assisted by VA-ECMO with local anaesthesia. EuroIntervention. 2025 Apr 7;21(7):e376-e378. doi: 10.4244/EIJ-D-24-00599. PMID: 40191880; PMCID: PMC11956023.

101. Pan C, Zhu Y, Zhao J, Zhang B, Hu S, Zhao C, et al. Prophylactic VA-ECMO During Complex High-Risk PCI: A Randomized Controlled Trial. JACC Adv. 2025 Sep;4(9):102095. doi: 10.1016/j.jacadv.2025.102095. Epub 2025 Aug 22. PMID: 40845741; PMCID: PMC12398820.

102. Den Uil CA, Daemen J, Lenzen MJ, Maugenest AM, Joziasse L, van Geuns RJ, Van Mieghem NM. Pulsatile iVAC 2L circulatory support in high-risk percutaneous coronary intervention. EuroIntervention. 2017 Feb 20;12(14):1689-1696. doi: 10.4244/EIJ-D-16-00371. PMID: 28216471.

103. Samol A, Schmidt S, Zeyse M, Wiemer M, Luani B. High-risk PCI under support of a pulsatile left ventricular assist device - First German experience with the iVAC2L system. Int J Cardiol. 2019 Dec 15;297:30-35. doi: 10.1016/j.ijcard.2019.10.020. Epub 2019 Oct 12. PMID: 31630819.

104. Bulum J, Bastos MB, Hlinomaz O, Malkin O, Pawlowski T, Dragula M, et al. Pulsatile Left Ventricular Assistance in High-Risk Percutaneous Coronary Interventions: Short-Term Outcomes. J Clin Med. 2024 Sep 10;13(18):5357. doi: 10.3390/jcm13185357. PMID: 39336843; PMCID: PMC11432136.
